# Purine Metabolism Profiling Reveals Distinct Associations of Uric Acid, Xanthine, and Hypoxanthine with Neurological Severity and Functional Outcome After Acute Ischemic Stroke

**DOI:** 10.3390/ijms27198486

**Published:** 2026-09-23

**Authors:** Iulian Roman-Filip, Lenard Farczadi, Corina Roman-Filip, Zoltan Bajko, Anca Moțățăianu, Smaranda Maier, Rodica Bălașa

**Affiliations:** 1 Doctoral School, ‘George Emil Palade’ University of Medicine, Pharmacy, Science, and Technology of Târgu Mureș, 540142 Targu Mures, Romania; 2 Chromatography and Mass Spectrometry Laboratory, Centre for Advanced Medical and Pharmaceutical Research, George Emil Palade University of Medicine, Pharmacy, Science, and Technology of Targu Mures, 540142 Targu Mures, Romania; 3 Department of Neurology, Faculty of Medicine, University “Lucian Blaga Sibiu”, 550024 Sibiu, Romania; 4 Neurology Department, University of Medicine, Pharmacy, Science and Technology “George Emil Palade” Targu Mures, 540142 Targu Mures, Romania

**Keywords:** acute ischemic stroke, purine metabolism, uric acid, xanthine, hypoxanthine, biomarkers

## Abstract

Purine metabolism and oxidative stress are increasingly recognized as important contributors to ischemic brain injury following acute ischemic stroke (AIS). Although uric acid has been extensively investigated because of its dual antioxidant and pro-oxidant properties, comparatively limited data exist regarding the prognostic relevance of upstream purine metabolites such as xanthine and hypoxanthine. The present study aimed to evaluate the association between serum uric acid, xanthine, and hypoxanthine concentrations and neurological severity and functional outcome following AIS. We performed a prospective observational cohort study including 143 consecutive patients admitted with acute ischemic stroke in a tertiary neurological center. Neurological severity was assessed using the National Institutes of Health Stroke Scale (NIHSS), while functional outcome was evaluated using the modified Rankin Scale (mRS) at discharge and at 3-month follow-up. Plasma xanthine and hypoxanthine concentrations were quantified using validated liquid chromatography–tandem mass spectrometry (LC–MS/MS) methodology. Distribution normality was assessed using Shapiro–Wilk and Kolmogorov–Smirnov testing. Comparative analyses were performed using Student’s *t*-test, the Mann–Whitney *U* test, and the Kruskal–Wallis test, according to the distribution and measurement scale of the variables. Correlations were assessed using Spearman’s rank correlation coefficient and visualized using a heatmap. Receiver operating characteristic (ROC) curve analysis and multivariable logistic regression models were additionally performed in order to evaluate the predictive performance of purine metabolism biomarkers for unfavorable functional outcome. Numerically lower serum uric acid concentrations were observed in patients with poorer functional outcomes at discharge and at 3 months, although these differences were not statistically significant. Hypoxanthine concentrations varied significantly according to admission NIHSS severity categories (*p* = 0.022), with lower values observed among patients presenting severe neurological deficits. Plasma xanthine concentrations demonstrated significant association with unfavorable long-term functional outcome (*p* = 0.033) and exhibited the highest predictive performance in ROC analysis (AUC = 0.614). In the adjusted multivariable logistic regression model, admission NIHSS remained the main independent predictor of unfavorable 3-month functional outcome, whereas xanthine and the other purine biomarkers did not retain statistically significant independent associations. Purine metabolism biomarkers showed exploratory associations with neurological severity and functional outcome, but the underlying biological mechanisms were not directly assessed. Simultaneous assessment of uric acid together with xanthine and hypoxanthine may provide additional insight into stroke severity and neurological recovery, supporting the potential role of purine metabolism profiling as a complementary biomarker approach in acute ischemic stroke.

## 1. Introduction

### 1.1. Acute Ischemic Stroke and Oxidative Stress

Acute ischemic stroke remains a leading cause of death and long-term disability despite advances in reperfusion therapy and secondary prevention [1].

Cerebral ischemia triggers metabolic, inflammatory, and oxidative processes that contribute to neuronal injury and functional impairment [2].

Although mechanical thrombectomy and intravenous thrombolysis have improved outcomes, recovery remains variable [3].

Hypoperfusion may cause energy depletion, mitochondrial dysfunction, excitotoxicity, and reactive oxygen species generation [4].

Reperfusion can further contribute to blood–brain barrier disruption, lipid peroxidation, inflammation, and neuronal apoptosis [5,6].

These processes have attracted increasing interest in research into endogenous antioxidant systems and circulating metabolic biomarkers as potential indicators of stroke severity and recovery [7,8,9].

### 1.2. Uric Acid as a Potential Neuroprotective Biomarker

Uric acid is the final product of human purine metabolism and has both antioxidant and vascular-risk-related properties [10].

Higher concentrations have been associated with endothelial dysfunction, inflammation, hypertension, and increased cerebrovascular risk [11,12].

Conversely, uric acid contributes to plasma antioxidant capacity and may reduce free-radical and peroxynitrite-mediated injury [13,14,15].

Experimental and clinical studies have investigated its potential effects on neuronal injury and reperfusion-related damage [16,17]. However, the relationship between serum uric acid and stroke outcome remains controversial, with studies reporting both possible neurological benefits and increased cardiovascular risk [18].

This variability may reflect differences in renal function, systemic inflammation, nutritional status, and other metabolic factors [19,20].

### 1.3. Hypoxanthine, Xanthine, and Purine Metabolism in Cerebral Ischemia

Compared with uric acid, the upstream purine metabolites hypoxanthine and xanthine have received less attention in ischemic stroke research [21].

ATP and adenine-nucleotide degradation produce hypoxanthine, which is subsequently converted to xanthine and uric acid through xanthine oxidase activity [22,23].

This pathway has been linked to reactive oxygen species generation and ischemia-related metabolic disturbance [24]. However, oxidative stress markers, ATP-related metabolites, and mitochondrial function were not directly measured in the present study; therefore, these mechanisms should be considered biological hypotheses rather than direct findings [25].

Previous studies have linked this enzymatic pathway to reactive oxygen species generation; however, these mechanisms were not directly assessed in the present study.

This strengthens the hypothesis that purine metabolites are indicators of oxidative stress severity and tissue hypoperfusion [26]. Elevated hypoxanthine and xanthine concentrations may indicate ischemia-related metabolic disruption and oxidative injury, supporting their potential as biomarkers of acute cerebral ischemia [27].

Prospective clinical studies evaluating the complete purine degradation pathway remain limited. Most previous research has focused on serum uric acid, while the clinical relevance of xanthine and hypoxanthine remains insufficiently established [28].

The present study therefore examined serum uric acid, xanthine, and hypoxanthine in relation to admission stroke severity, neurological evolution, and functional outcome at discharge and three months.

**Figure 1:**
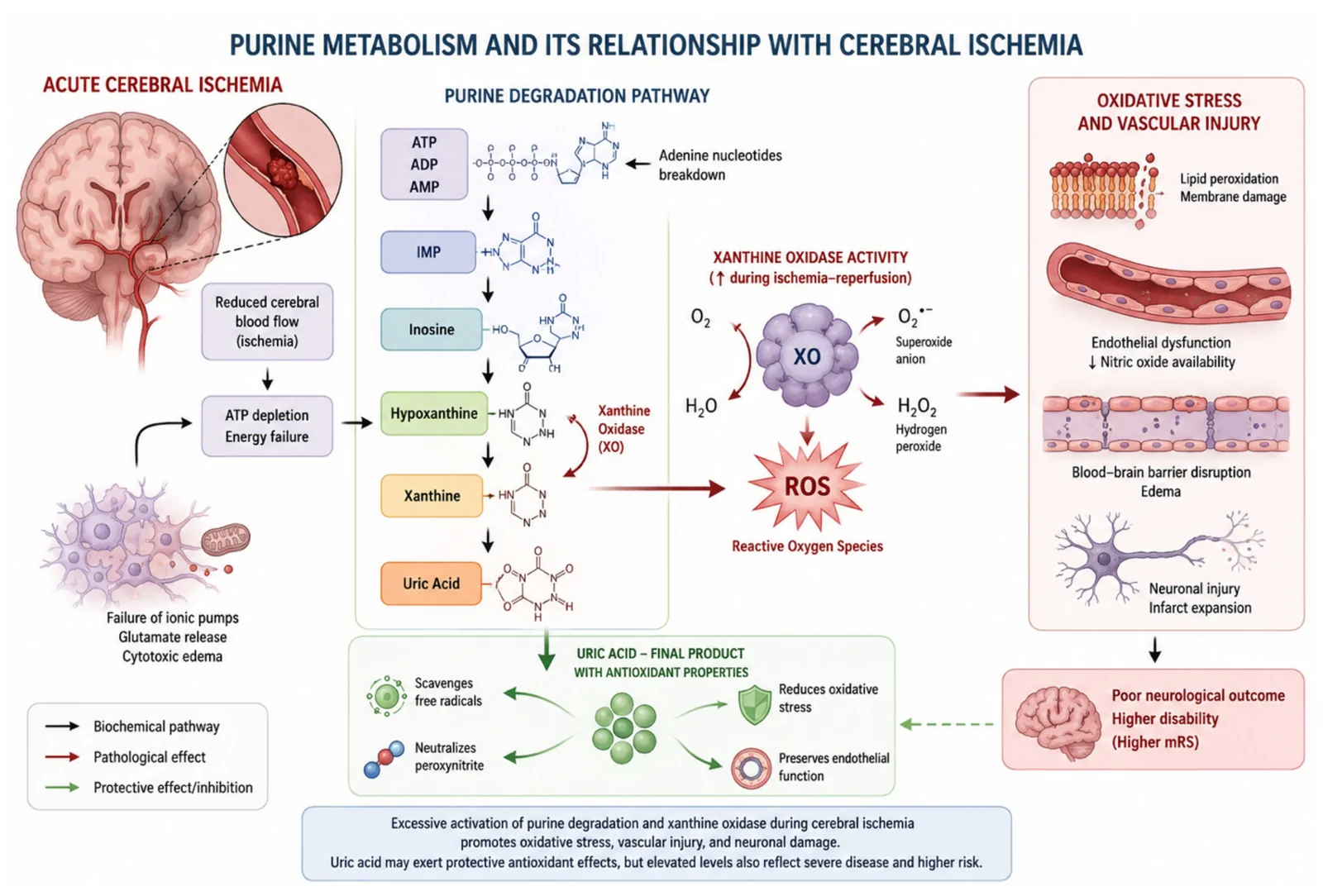
**Proposed Relationship Between Purine Metabolism and Oxidative Stress in Acute Ischemic Stroke.** Schematic illustration of the proposed role of purine metabolism during acute cerebral ischemia. Reduced cerebral blood flow leads to ATP depletion and increased formation of hypoxanthine, xanthine, and uric acid. During this process, activation of xanthine oxidase contributes to the generation of reactive oxygen species (ROS), promoting oxidative stress, endothelial dysfunction, blood–brain barrier injury, and neuronal damage [29]. In experimental models, uric acid has been reported to attenuate oxidative injury through suppression of reactive oxygen species accumulation, lipid peroxidation, and related oxidative damage pathways [30]. The purpose of this figure was to summarize the potential interactions between energetic failure caused by ischemia and purine metabolic alterations and neurological outcome following a cerebrovascular event. The figure presents a proposed biological framework based on the previous literature and does not represent mechanisms directly measured in the present study.

### 1.4. Nutritional Relevance of Uric Acid and Purine Metabolism

Serum uric acid concentrations are influenced by dietary intake and metabolic status, including purine-rich foods, fructose, alcohol, protein intake, and renal function [31,32].

**Dietary patterns may also influence oxidative stress and cardiovascular health, although detailed nutritional assessment was not performed in the present study. In particular, excessive fructose consumption has been associated with oxidative stress, hyperuricemia, metabolic dysfunction, and adverse cardiovascular effects** [33,34,35,36].

These factors should therefore be considered when interpreting circulating purine biomarkers [37,38].

### 1.5. Experimental Evidence Supporting the Neuroprotective Role of Uric Acid

Experimental studies have reported possible antioxidant and neuroprotective effects of uric acid, including reduced infarct size and improved blood–brain barrier integrity after ischemic injury [39,40,41].

A schematic overview of the role of oxidative stress in ischemic stroke is presented in Figure 1.

Uric acid has also been investigated in combination with reperfusion therapy, but clinical findings remain inconsistent and require further evaluation [42,43].

### 1.6. Study Rationale and Objectives

Although uric acid has been widely investigated, prospective studies evaluating uric acid together with upstream metabolites such as xanthine and hypoxanthine remain limited [44].

This prospective observational study examined the associations of serum uric acid, xanthine, and hypoxanthine with admission stroke severity, neurological evolution, and functional outcomes at discharge and three months. The study aimed to assess their potential clinical relevance while recognizing that nutritional status, oxidative stress, and mitochondrial function were not directly measured.

## 2. Results

The principal findings of the present study are summarized in Table 1, Table 2, Table 3, Table 4, Table 5, Table 6, Table 7, Table 8 and Table 9, which provide the detailed demographic, clinical, biochemical, and statistical results.

**Table 6 comparison of Serum Uric Acid Concentration According to Admission NIHSS Severity.** Patients were stratified by admission NIHSS score into mild stroke (NIHSS ≤ 4) and moderate-to-severe stroke (NIHSS > 4) groups. Serum uric acid concentration was analyzed as a continuous variable. Normality testing with the Shapiro–Wilk test showed a non-normal distribution in at least one subgroup (mild stroke *p* = 0.234; moderate-to-severe stroke *p* = 0.063); therefore, a non-parametric Mann–Whitney U test was used for comparison. Continuous variables are presented as mean ± standard deviation (SD), along with the median and minimum–maximum ranges. No statistically significant difference in serum uric acid concentration was observed between NIHSS severity groups (*p* = 0.289).

ROC analysis showed modest discriminatory performance for all three biomarkers. Xanthine had the highest AUC (0.614), whereas uric acid and hypoxanthine had AUCs of 0.506 and 0.477, respectively. We also acknowledge this as a limitation. These values indicate limited standalone discrimination for predicting poor 3-month functional outcome.

For the analyses of prognostic performance, patients were classified according to three-month functional outcome as favorable (mRS 0–3) or unfavorable (mRS 4–6). The ROC analysis and multivariable logistic regression model used this same outcome definition.

### Overall Interpretation of Results

The present prospective cohort included 143 patients with acute ischemic stroke. Serum uric acid showed a relatively symmetrical distribution, whereas xanthine and hypoxanthine demonstrated right-skewed distributions with substantial interindividual variability. These distributional characteristics were considered when selecting the statistical methods for the comparative and regression analyses.

Serum uric acid was not significantly different between the predefined admission NIHSS severity groups (*p* = 0.289). Numerically lower serum uric acid concentrations were observed among patients with poorer functional outcomes at discharge and at 3 months; however, these differences across mRS categories were not statistically significant.

In the ROC analysis, xanthine demonstrated the highest AUC among the evaluated biomarkers (AUC = 0.614), followed by uric acid (AUC = 0.506) and hypoxanthine (AUC = 0.477) (Figure 2). These AUC values indicate modest discriminatory performance.

The standardized biomarker profiles according to 3-month functional outcome are presented in Figure 3. Overall, the biomarkers demonstrated distinct distribution patterns between favorable and unfavorable outcome groups, although these descriptive profiles should be interpreted together with the corresponding statistical analyses.

The correlation analysis showed positive relationships among admission NIHSS, NIHSS at 7 days, mRS at discharge, and mRS at 3 months. Serum uric acid showed a positive correlation with creatinine, whereas xanthine and hypoxanthine generally showed weak correlations with conventional metabolic and lipid variables (Figure 4). These findings describe the observed relationships within the cohort but do not establish the underlying biological mechanisms.

Hypoxanthine concentrations differed significantly across the admission NIHSS severity categories (Kruskal–Wallis test, *p* = 0.022; Figure 5). Median concentrations were 1249.36 ng/mL in the mild group, 865.48 ng/mL in the moderate group, and 843.09 ng/mL in the severe group. The severe-stroke subgroup was relatively small; therefore, these findings should be interpreted cautiously.

Xanthine concentrations did not differ significantly across the admission NIHSS severity categories (*p* = 0.145). In contrast, xanthine concentrations differed significantly between the predefined 3-month functional-outcome groups, with higher concentrations observed among patients with unfavorable outcome (Mann–Whitney U test, *p* = 0.033; Figure 6).

In the adjusted multivariable logistic regression model, poor functional outcome was defined as an mRS score of 4–6 at 3 months (Figure 7). The model included admission NIHSS, serum uric acid, log-transformed xanthine, log-transformed hypoxanthine, age, sex, hypertension, LDL cholesterol, and HDL cholesterol. Admission NIHSS was associated with poor outcome (OR 1.20 per point increase, 95% CI 1.10–1.32; *p* < 0.001), and age was also associated with poor outcome (OR 1.06 per year increase, 95% CI 1.02–1.11; *p* = 0.004). The adjusted associations for serum uric acid, xanthine, hypoxanthine, sex, hypertension, LDL cholesterol, and HDL cholesterol were not statistically significant.

In the adjusted multivariable logistic regression model, poor functional outcome was defined as an mRS score of 4–6 at 3 months. The model included admission NIHSS, serum uric acid, log-transformed xanthine, log-transformed hypoxanthine, age, sex, hypertension, LDL cholesterol, and HDL cholesterol. Admission NIHSS was associated with poor outcome (OR 1.20 per point increase, 95% CI 1.10–1.32; *p* < 0.001), and age was also associated with poor outcome (OR 1.06 per year increase, 95% CI 1.02–1.11; *p* = 0.004). The adjusted associations for serum uric acid, xanthine, hypoxanthine, sex, hypertension, LDL cholesterol, and HDL cholesterol were not statistically significant. The model was based on 136 complete cases and should be interpreted cautiously because of the moderate sample size and limited number of outcome events.

Overall, the three biomarkers demonstrated distinct patterns across the evaluated clinical measures. Hypoxanthine differed across admission NIHSS categories, whereas xanthine differed between the predefined 3-month outcome groups and showed the highest AUC among the evaluated biomarkers. Serum uric acid showed numerical differences across functional outcome groups, but these differences were not statistically significant. Nevertheless, the discriminatory performance of the individual biomarkers was modest, and the observational design does not permit causal conclusions. The biological interpretation of these findings should therefore be considered hypothesis-generating and requires confirmation in larger, adequately powered, multicenter studies with standardized sampling times and serial biomarker measurements.

## 3. Discussions

### 3.1. General Discussions

The pathophysiology of ischemic stroke involves cytotoxicity, mitochondrial impairment, oxidative stress, inflammation, endothelial injury, and metabolic dysfunction [55].

Purines and their metabolites have attracted interest because of their involvement in ATP degradation and oxidative processes during ischemic injury [56].

In this prospective observational study, serum uric acid and the upstream purine metabolites xanthine and hypoxanthine were evaluated in relation to neurological severity and functional outcomes following acute ischemic stroke. The findings indicate heterogeneous associations between individual purine metabolites, stroke severity, and clinical evolution, as shown in Table 1, Table 2, Table 3, Table 4, Table 5, Table 6 and Table 7.

As shown in Table 8, no statistically significant differences in serum uric acid, xanthine, or hypoxanthine concentrations were identified across TOAST etiological subtypes. Although some numerical variability was observed between subgroups, these differences did not reach statistical significance and should be interpreted cautiously given the subgroup sample sizes. Therefore, the present data do not support a clear association between purine biomarker concentrations and stroke etiology as classified by TOAST.

A recent systematic review reported variable prognostic significance of uric acid in cerebral ischemia, with studies suggesting both potential neurological benefits and increased cardiovascular risk [57].

Uric acid has also been associated with hypertension and other vascular risk factors that may contribute to cerebrovascular morbidity [58].

Some hemorrhagic patterns that lack apparent causes may also be associated with the negative metabolic climate created by the high concentration of this compound [59,60].

In the present cohort, numerically lower serum uric acid concentrations were observed among patients with poorer functional outcomes at discharge and after three months; however, these differences were not statistically significant. Previous experimental and clinical studies have proposed antioxidant properties of uric acid; however, the present study did not demonstrate statistically significant differences across functional outcome categories and cannot establish a causal or neuroprotective effect [61].

Previous studies have reported associations between higher acute-phase uric acid concentrations and improved neurological outcomes; however, the present study did not demonstrate statistically significant differences across functional outcome categories [62].

The numerical trends observed in the functional outcome analyses and ROC analysis (Figure 3, Figure 4, Figure 5, Figure 6 and Figure 7) should therefore be interpreted cautiously. The potential role of uric acid during reperfusion and oxidative injury remains an area of ongoing investigation.

Experimental studies have suggested that uric acid may reduce free-radical-mediated reperfusion injury and support endothelial or blood–brain barrier integrity during oxidative stress [63,64]. Nevertheless, the relationship between uric acid and ischemic stroke remains controversial [65].

Hyperuricemia has been associated with hypertension, diabetes mellitus, chronic kidney disease, metabolic syndrome, endothelial dysfunction, and increased long-term cardiovascular risk [66,67].

Accordingly, uric acid may reflect both vascular risk burden and an endogenous antioxidant response during acute ischemia [68,69].

The multivariable analysis showed that admission NIHSS and age remained independently associated with poor functional outcome, whereas the associations for serum uric acid, xanthine, and hypoxanthine were not statistically significant after adjustment.

Although uric acid has been extensively studied, clinical investigations of upstream purine metabolites such as xanthine and hypoxanthine remain limited [70]. ATP degradation produces hypoxanthine, which is subsequently converted to xanthine and uric acid through xanthine oxidase activity. Importantly, this enzymatic pathway is closely linked to reactive oxygen species production and oxidative cellular injury [71].

Elevated hypoxanthine and xanthine concentrations may therefore indicate ischemia-related metabolic disruption, but they should not be considered direct measurements of oxidative stress in the present study [72].

Table 9 summarizes the metabolic and purine-related characteristics of the cohort. These descriptive data provide clinical context for interpreting the biomarker analyses but do not directly demonstrate oxidative stress, ischemic injury mechanisms, or causal associations. The prevalence of hypertension, dyslipidemia, and impaired glycemic status reflects the cardiometabolic burden commonly observed in patients with acute ischemic stroke [73].

The relevance of these findings to other stroke mechanisms, including cervical artery dissection, remains uncertain and was not directly investigated in this cohort [74].

Lower hypoxanthine concentrations were observed among patients with more severe admission NIHSS scores. This finding may reflect differences in sampling time, disease severity, systemic metabolism, renal clearance, or downstream metabolite conversion. These explanations remain hypothetical because mitochondrial function, ATP-related metabolites, and direct oxidative stress markers were not measured [75,76].

The observed association between hypoxanthine concentrations and admission NIHSS severity may reflect ischemia-related ATP degradation and subsequent purine-metabolite conversion rather than a direct causal effect of hypoxanthine. The relationship may also be influenced by the severity and duration of ischemia, sampling time, reperfusion status, renal clearance, and systemic metabolic conditions. Because ATP-related metabolites, oxidative-stress markers, and mitochondrial function were not directly measured, these mechanisms remain plausible interpretations rather than demonstrated biological pathways.

Xanthine showed the strongest association with unfavorable three-month functional outcome and the highest discriminatory performance among the evaluated biomarkers. However, its AUC was modest, and its performance remained below the level generally considered acceptable for standalone clinical prediction. In the multivariable model, the associations of the biomarkers were evaluated after adjustment for available clinical and biochemical variables. These findings support the potential value of xanthine as a complementary biomarker, but not as an independent diagnostic or prognostic tool [77,78].

The correlation heatmap showed relatively weak associations between xanthine, hypoxanthine, and conventional lipid or metabolic variables, whereas stronger correlations were observed between neurological severity and functional outcome measures. These findings provide descriptive information about the relationships among the measured variables but do not establish distinct mitochondrial or oxidative mechanisms.

Purine-metabolite concentrations may also be influenced by dietary purine intake, renal clearance, systemic metabolic status, hydration, medication exposure, and antioxidant reserve [79].

Detailed nutritional assessment, eGFR, hydration status, diuretic use, serial sampling, oxidative stress markers, ATP-related metabolites, and direct measures of mitochondrial function were not systematically available. Therefore, residual confounding cannot be excluded, and the proposed relationships between purine metabolites, oxidative stress, ATP depletion, and mitochondrial dysfunction should be considered hypotheses for future investigation.

Previous experimental and clinical studies have suggested possible neuroprotective effects of uric acid during ischemia and reperfusion [80,81,82,83]. However, these findings cannot be directly extrapolated to the present observational cohort because exogenous uric acid administration was not evaluated.

An important strength of this study was the simultaneous assessment of uric acid, xanthine, and hypoxanthine using validated LC–MS/MS methodology. Most previous clinical studies have focused mainly on serum uric acid [84].

The prospective design also enabled predefined neurological and functional assessments during hospitalization and follow-up. Nevertheless, the single-center design, moderate sample size, incomplete control of nutritional and renal confounding, modest predictive performance, missing values, and absence of external validation limit the generalizability of the findings. Overall, the results support further investigation of purine metabolites as complementary biomarkers in acute ischemic stroke, while their independent clinical utility and underlying mechanisms require confirmation.

From a translational perspective, modulation of purine metabolism, including uric acid supplementation, remains an investigational strategy rather than an established treatment for acute ischemic stroke. Although uric acid may provide antioxidant effects under selected ischemic or reperfusion conditions, supplementation could also carry potential risks related to hyperuricemia, vascular inflammation, renal impairment, and cardiovascular or cerebrovascular events. Therefore, any therapeutic application would require careful patient selection, dose evaluation, and confirmation in adequately powered randomized clinical trials [85].

### 3.2. Limitations

Several limitations should be acknowledged. First, this was a single-center observational study with a relatively moderate sample size, particularly for subgroup analyses, and no formal a priori power calculation was performed. Therefore, the study may have had limited power to detect small or subgroup-specific associations. Second, xanthine and hypoxanthine showed substantial interindividual variability, right-skewed distributions, and several outlying values. These characteristics were addressed using non-parametric analyses and logarithmic transformation where appropriate.

Third, biomarkers were measured only once during the acute phase. Therefore, temporal changes in purine metabolism during hospitalization and recovery could not be assessed, and serial sampling may provide additional information about the dynamic relationship between purine metabolites and clinical evolution.

Fourth, several potential confounders could not be fully controlled. Additional variables related to metabolic status, lifestyle exposures, renal function, nutritional intake, hydration, and medication use, including diabetes mellitus, smoking status, alcohol consumption, eGFR, dietary purine intake, hydration status, diuretic use, and other relevant medication exposure, were considered during model specification. However, these variables were incompletely documented, non-uniformly recorded, or unavailable at sufficient granularity across the cohort to permit reliable incorporation into the adjusted multivariable model without reducing model stability. Therefore, residual confounding from these factors cannot be excluded.

Fifth, although the total cohort comprised 143 patients, the number of observations varied between variables because of incomplete documentation, unavailable routine laboratory results, or insufficient biological samples for specialized LC–MS/MS measurements. Available sample sizes were therefore reported separately for each variable, including age (n = 143), HDL-cholesterol (n = 141), triglycerides (n = 137), xanthine (n = 136), and hypoxanthine (n = 132). These differences may have reduced the effective sample size of selected analyses and may have introduced selection bias.

Sixth, the discriminatory performance of the individual biomarkers was modest, with the highest AUC observed for xanthine (0.614), below the level generally considered acceptable for standalone clinical prediction. Accordingly, these biomarkers should not be interpreted as independent diagnostic or prognostic tools. In addition, the incremental predictive value of purine metabolites beyond established clinical predictors was not formally quantified.

Finally, oxidative-stress markers, ATP-related metabolites, and direct measures of mitochondrial function were not collected. Therefore, the proposed relationships between purine metabolites, oxidative stress, ATP depletion, and mitochondrial dysfunction remain hypothetical. External validation was not performed because an independent validation cohort was unavailable. The findings should therefore be considered exploratory and hypothesis-generating.

### 3.3. Final Considerations

Despite these limitations, the present study supports the growing concept that purine metabolism may represent an important pathophysiological component of acute cerebral ischemia [86]. Simultaneous assessment of uric acid, xanthine, and hypoxanthine provides a more complex characterization of oxidative metabolic response during acute stroke and may contribute to improved understanding of ischemia-related biochemical alterations. Future multicenter prospective studies including serial biomarker measurements, external validation cohorts, nutritional and metabolic assessment, and integration with advanced neuroimaging parameters are required to further clarify the prognostic and mechanistic significance of purine metabolism disturbances in ischemic stroke.

Purine-metabolite profiling may provide complementary information in acute ischemic stroke, although its current clinical implementation is limited by assay availability, cost, and the need for specialized LC–MS/MS facilities. Further multicenter studies should establish analytical standardization, clinical utility, and cost-effectiveness before routine application can be considered.

## 4. Materials and Methods

### 4.1. Study Design and Population

This study was designed as a prospective observational cohort study conducted at Târgu Mureș University County Hospital, Târgu Mureș, Romania, a tertiary referral center specialized in the management of acute cerebrovascular pathology. Consecutive patients admitted with acute ischemic stroke were prospectively enrolled over the study period and systematically evaluated using predefined clinical, laboratory, and imaging protocols. The prospective design was selected in order to ensure standardized data collection, accurate temporal assessment of biomarker dynamics, and consistent evaluation of neurological and functional outcomes during both hospitalization and follow-up.

A prospective methodology was considered particularly important given the dynamic metabolic alterations occurring during acute cerebral ischemia. Purine metabolism biomarkers such as uric acid, hypoxanthine, and xanthine may be significantly influenced by the timing of sampling, acute reperfusion therapies, neurological deterioration, systemic inflammatory response, renal function, and metabolic status. Prospective enrollment therefore allowed for controlled and uniform acquisition of blood samples and clinical parameters during the acute phase of stroke, reducing recall bias and minimizing inconsistencies frequently encountered in retrospective analyses.

The sample size was determined by the number of consecutive eligible patients recruited during the predefined study period. No formal a priori power calculation was performed. This is also stated in the Limitations subsection.

Patients were prospectively and consecutively enrolled from eligible admissions during the study period. No selective recruitment based on biomarker concentrations, stroke severity, or clinical outcome was performed.

Furthermore, the prospective design enabled sequential neurological and functional assessment using standardized scales applied at predefined time points, including the National Institutes of Health Stroke Scale (NIHSS) [87] at admission and during hospitalization, as well as the modified Rankin Scale (mRS) [88] at discharge and three-month follow-up. This longitudinal approach permitted evaluation not only of baseline stroke severity but also of short-term neurological evolution and functional recovery, allowing for a more comprehensive characterization of the relationship between purine metabolism biomarkers and clinical outcome.

All patients underwent diagnostic evaluation according to current stroke management guidelines, including neurological examination, cerebral imaging, vascular assessment, and routine laboratory investigations. Acute ischemic stroke diagnosis was established based on compatible clinical presentation and neuroimaging findings. Demographic characteristics, vascular risk factors, stroke subtype, reperfusion therapies, and laboratory parameters were prospectively recorded in a dedicated institutional database.

The inclusion criteria were as follows:

1. Adult patients aged ≥ 18 years;
2. Admission for acute ischemic stroke confirmed clinically and with imaging;
3. Availability of complete neurological assessment including NIHSS evaluation at admission;
4. Availability of follow-up neurological and functional evaluation during hospitalization and/or at three months;
5. Serum determination of uric acid, hypoxanthine, and xanthine performed during the acute phase of stroke;
6. Availability of routine laboratory investigations including renal and metabolic parameters;
7. Completion of standard etiological and vascular diagnostic workup for ischemic stroke;
8. Signed informed consent obtained from the patient or legally authorized representative, when applicable.

The exclusion criteria were as follows:

1. Intracerebral hemorrhage or other non-ischemic cerebrovascular pathology;
2. Transient ischemic attack without imaging-confirmed cerebral infarction;
3. Stroke mimics or uncertain cerebrovascular diagnosis;
4. Active malignant disease with systemic involvement;
5. Severe acute infectious or inflammatory disorders potentially influencing oxidative stress biomarkers;
6. Advanced renal insufficiency or end-stage renal disease significantly affecting purine metabolism and uric acid clearance;
7. Known hematological or metabolic disorders influencing purine metabolism;
8. Recent major surgery or severe systemic trauma;
9. Incomplete clinical, laboratory, or follow-up data;
10. Inability to obtain adequate biochemical samples for purine metabolite analysis.
11. Current or previous use of medications capable of significantly influencing purine metabolism or lowering serum uric acid concentrations, including urate-lowering therapies and xanthine oxidase inhibitors.

Participants with advanced renal insufficiency, end-stage renal disease, known metabolic disorders affecting purine metabolism, or current use of urate-lowering or xanthine-oxidase-inhibiting medication were excluded according to the predefined eligibility criteria. However, residual confounding related to renal function, nutrition, hydration, and other medications cannot be excluded because these factors were not systematically measured or included in the adjusted model.

Stroke etiology was classified according to the Trial of Org 10172 in Acute Stroke Treatment (TOAST) criteria [89], while clinical severity and functional outcome were assessed using validated neurological scales [90]. Reperfusion therapies, including intravenous thrombolysis and mechanical thrombectomy, were performed according to contemporary international recommendations and institutional protocols [91].

The study protocol was approved by the institutional ethics committee, and all procedures were conducted in accordance with the principles of the Declaration of Helsinki [92]. Informed consent for participation and data collection was obtained from patients or legally authorized representatives whenever applicable.

#### 4.1.1. Blood Sampling and Pre-Analytical Handling

Blood samples were collected within 24 h of admission for the respective cerebrovascular event. Before processing, samples were stored under temperature-controlled refrigerated conditions at 4–5 °C for up to 120 min. Fasting status was not consistently documented because sampling was performed during the acute phase of hospitalization. Only a limited number of patients received reperfusion therapy; however, the exact timing of blood collection in relation to thrombolysis or thrombectomy was not systematically recorded. Sampling was therefore standardized to the admission assessment rather than to a fixed interval from symptom onset or reperfusion treatment.

Because blood collection occurred within the emergency-care workflow, exact symptom-onset or last-known-well times were not consistently available for all patients. Differences in triage procedures, emergency response times, and the clinical condition at presentation may have limited precise documentation of the interval between symptom onset and sampling. In addition, a standardized electronic system for recording these time points was not available. This potential variability was considered when interpreting the biomarker results.

Plasma samples were processed according to the validated CROMS laboratory protocol. For each analysis, 150 μL of plasma was mixed with 10 μL of isotopically labelled xanthine-^13^C,^15^N_2_ internal standard and vortexed for 1 min at 2000 rpm. Subsequently, 450 μL of acetonitrile was added for protein precipitation, followed by vortex mixing for an additional 1 min at 2000 rpm. Samples were centrifuged at 10,000 rpm for 5 min. A 500 μL aliquot of the supernatant was transferred to clean tubes and evaporated to dryness under vacuum for approximately 6 h at room temperature. The dried extracts were reconstituted in 500 μL of 50 mM ammonium hydroxide in water (95:5, *v*/*v*), mixed for 1 h at 800 rpm, and transferred to HPLC vials. The prepared samples were introduced into the autosampler immediately after preparation.

Chromatographic separation was performed using a Gemini NX-C18 column (3.0 × 100 mm, 3 μm) maintained at 20 °C, with the autosampler maintained at 25 °C. Xanthine and hypoxanthine were quantified using liquid chromatography–tandem mass spectrometry with positive electrospray ionization and multiple-reaction monitoring. Six-point calibration curves were prepared over the concentration range of 5–1000 ng/mL for both analytes, with a lower limit of quantification of 5 ng/mL.

**Note:** The exact interval between symptom onset (or last-known well time) and blood collection, as well as the precise timing of sampling in relation to reperfusion treatment, was not consistently available. No freeze–thaw cycles were performed before analysis, as samples were processed according to the laboratory protocol. Patients receiving urate-lowering therapy or other medications known to substantially affect purine metabolism were excluded where documented.

#### 4.1.2. Patient Enrollment and Follow-Up

A total of 183 consecutive patients with suspected acute ischemic stroke were assessed for eligibility. Forty patients were excluded: 17 because of previous urate-lowering therapy or medications affecting purine metabolism, 11 because of incomplete laboratory sampling, 5 because of declined participation, 5 because of hemorrhagic stroke, and 2 because of stroke mimics or subsequently confirmed myelopathy. No additional patients were excluded for failure to meet the predefined inclusion criteria. Consequently, 143 patients were prospectively enrolled. All enrolled participants completed the 3-month follow-up, resulting in no loss to follow-up. The complete-case adjusted multivariable regression analysis included 136 patients; 7 patients were excluded from this model because of incomplete data for one or more model variables (Figure 8).

Blood samples for serum uric acid and plasma xanthine and hypoxanthine measurements were collected at hospital admission, during the initial diagnostic evaluation for acute ischemic stroke. The admission NIHSS assessment and routine clinical and laboratory investigations were performed at the same baseline evaluation. Samples were collected before or as close as possible to the initiation of acute stroke treatment, according to the patient’s clinical condition and the requirements of emergency management. The exact time from symptom onset to blood collection and the timing in relation to reperfusion therapy were not systematically recorded for all participants; therefore, the biomarkers were interpreted as admission-phase measurements rather than as precisely timed measurements from symptom onset.

Purine-metabolite concentrations were measured once during the acute phase of hospitalization. Serial sampling was not performed because repeated specialized LC–MS/MS measurements were not routinely feasible within the available laboratory resources and the emergency-care setting, where discharge schedules and patient turnover were highly variable.

#### 4.1.3. Clinical and Functional Outcome Assessment

Neurological severity was assessed using the NIHSS at admission and during hospitalization, whereas functional outcome was assessed using the mRS at hospital discharge and at three-month follow-up. Assessments were performed as part of routine clinical care; formal assessor blinding was not implemented.

### 4.2. Statistical Analysis

Statistical analysis was performed to evaluate the relationship between purine metabolism biomarkers and both neurological severity and functional outcome following acute ischemic stroke. Continuous variables were initially assessed descriptively and are presented as mean ± standard deviation (SD), median values, and minimum–maximum ranges according to data distribution characteristics. Categorical variables were expressed as absolute frequencies and percentages.

Prior to comparative statistical analysis, distribution normality was evaluated using the Shapiro–Wilk test and Kolmogorov–Smirnov test because of its high sensitivity for detecting deviations from Gaussian distribution in moderate-sized clinical datasets. Variables demonstrating normal distribution were subsequently analyzed using parametric statistical methods, while variables exhibiting non-Gaussian distribution, marked right-skewness, or extreme outlier values were analyzed using non-parametric methodology.

The adjusted model included the available potential confounders: age, sex, hypertension, LDL-cholesterol, HDL-cholesterol, admission NIHSS, serum uric acid, log-transformed xanthine, and log-transformed hypoxanthine. eGFR, dietary purine intake, hydration status, and diuretic use were not systematically available and were therefore not included.

Additional potential confounders, including diabetes mellitus, smoking status, and alcohol consumption, were considered during model specification. However, these variables were not recorded with sufficient completeness, uniformity, or granularity across the cohort to support reliable inclusion in the adjusted multivariable model without substantially reducing the effective sample size or introducing additional instability. Similarly, eGFR, dietary purine intake, hydration status, and diuretic use were incompletely documented and could not be incorporated consistently. Therefore, although the model adjusted for the available demographic, clinical-severity, vascular-risk, lipid, and biomarker variables, residual confounding related to metabolic status, lifestyle exposures, renal function, nutritional factors, hydration, and medication use cannot be excluded.

Comparisons between two independent groups were performed using Student’s *t*-test for approximately normally distributed continuous variables and the Mann–Whitney *U* test for non-normally distributed variables. Comparisons involving three or more independent groups were performed using the Kruskal–Wallis test. Functional outcomes were assessed using mRS scores at discharge and at 3 months, while neurological severity was assessed using NIHSS scores at admission and during hospitalization.

Unfavorable functional outcome at 3-month follow-up was consistently defined as an mRS score of 4–6, corresponding to mRS > 3, and this definition was applied in both the ROC and multivariable logistic regression analyses. This threshold was selected to identify patients with clinically relevant dependence in mobility and daily activities. The definition was applied consistently in the ROC analysis and multivariable logistic regression model.

Because xanthine and hypoxanthine concentrations demonstrated substantial variability between patients and a markedly skewed distribution with several extreme values, logarithmic scaling was applied in selected figures and regression analyses in order to improve data visualization and reduce the influence of outlier values. Correlation analysis between clinical, biochemical, and functional parameters was performed using Spearman rank correlation coefficients because several analyzed variables did not demonstrate normal distribution. The resulting correlations were subsequently illustrated using a heatmap representation in order to provide an integrated overview of the relationships between neurological severity, functional outcome, metabolic parameters, and purine metabolism biomarkers.

Receiver operating characteristic (ROC) curve analysis was performed to evaluate the ability of serum uric acid, xanthine, and hypoxanthine concentrations to identify unfavorable functional outcome at 3-month follow-up, consistently defined as mRS 4–6, corresponding to mRS > 3. The discriminatory performance of each biomarker was assessed by calculating the area under the curve (AUC).

Adjusted multivariable regression analysis was performed to assess the associations between the included predictors and poor 3-month functional outcome. Poor outcome was defined as an mRS score of 4–6. The model included admission NIHSS, serum uric acid, log-transformed xanthine, log-transformed hypoxanthine, age, sex, hypertension, LDL cholesterol, and HDL cholesterol. Xanthine and hypoxanthine were logarithmically transformed because of their right-skewed distributions. Odds ratios with corresponding 95% confidence intervals were calculated and presented in the forest plot. The analysis included 136 complete cases.

Multivariable logistic regression was used to evaluate whether xanthine, hypoxanthine, and uric acid remained associated with unfavorable three-month functional outcome after adjustment for available clinical predictors, including age, admission NIHSS, sex, hypertension, LDL-cholesterol, and HDL-cholesterol. A formal assessment of incremental predictive value, such as change in AUC, likelihood-ratio testing, or net reclassification analysis, was not performed.

All statistical analyses were performed using 3.12.7 (Python Software Foundation, Wilmington, DE, USA) , with data management conducted using pandas, statistical testing using SciPy library (version 1.14.1), and multivariable logistic regression and variance inflation factor analyses using Statsmodels package (version 0.14.4).A two-sided *p*-value < 0.05 was considered statistically significant throughout all analyses. Given the exploratory and observational nature of the study, statistical findings were interpreted cautiously, particularly in subgroup analyses characterized by limited sample size or substantial biomarker variability.

### 4.3. Quantification of Xanthine and Hypoxanthine by LC–MS/MS

Quantitative determination of plasma xanthine and hypoxanthine concentrations was performed using a validated liquid chromatography–tandem mass spectrometry (LC–MS/MS) method [93] developed within the CROMS Laboratory, Center for Advanced Medical and Pharmaceutical Research.

The method was optimized for quantitative assessment of purine metabolism intermediates in human plasma and included standardized procedures for sample preparation, chromatographic separation, calibration, and analytical validation.

Plasma samples were processed on the day of analysis. Briefly, 150 µL of plasma was mixed with isotopically labeled xanthine-^13^C,^15^N_2_ internal standard solution, followed by protein precipitation using acetonitrile. After vortex homogenization and centrifugation, the supernatant was collected, evaporated to dryness under vacuum, and reconstituted in ammonium solution: methanol mixture prior to LC–MS/MS analysis.

Perkin Elmer Chromatographic separation was performed using a Gemini NX-C18 analytical column (Phenomenex, Torrance, CA, USA) coupled to a Flexar FX-10 UHPLC system (PerkinElmer, Waltham, MA, USA). The mobile phase consisted of aqueous formic acid and methanol containing formic acid, delivered at a flow rate of 0.400 mL/min. Total run time was 5 min per sample. Mass spectrometric detection was performed using a QTOF 4600 mass spectrometer (AB Sciex, USA) operating in positive electrospray ionization mode (ESI+). Quantitative analysis was conducted in multiple reaction monitoring (MRM) mode using analyte-specific precursor/product ion transitions for xanthine and hypoxanthine together with isotopically labeled internal standards [94].

Calibration curves were generated using six concentration levels ranging from 5 to 1000 ng/mL and demonstrated excellent linearity across the analytical range. The lower limit of quantification (LLOQ) for both analytes was 5 ng/mL without detectable carry-over effects. Method validation included evaluation of within-run and between-run precision, analytical recovery, and accuracy using predefined quality control concentrations. Mean analytical recovery exceeded 98% for both analytes, while precision and accuracy values remained within accepted validation limits throughout the analytical range. All of the measurements were performed in conformity with international standards [95].

The validated LC–MS/MS method enabled sensitive and reproducible quantification of plasma xanthine and hypoxanthine concentrations and provided the analytical basis for subsequent correlation with neurological severity and functional outcome in patients with acute ischemic stroke [96].

Representative LC–MS/MS chromatograms demonstrating the chromatographic separation and quantitative detection of xanthine and hypoxanthine are presented in Figure 9.

## 5. Conclusions

In conclusion, the present study suggests that purine metabolism may represent an important component of the complex biochemical response occurring during acute ischemic stroke. By simultaneously evaluating serum uric acid together with its precursor metabolites, xanthine and hypoxanthine, we attempted to obtain a broader perspective on the relationship between oxidative stress, energy failure, and neurological recovery following cerebral ischemia.

Our results showed numerically lower serum uric acid concentrations among patients with less favorable functional outcomes; however, these differences were not statistically significant. Although this numerical trend is consistent with hypotheses regarding the antioxidant properties of uric acid proposed in previous studies, the present findings do not establish an independent prognostic association or a causal neuroprotective effect.

At the same time, xanthine showed the highest ROC AUC among the analyzed purine biomarkers and was associated with unfavorable 3-month functional outcome in unadjusted analysis; however, this association was not statistically significant in the adjusted multivariable model. Hypoxanthine concentrations differed across admission NIHSS severity categories, but their independent prognostic significance was not confirmed.

In this cohort, purine biomarkers showed limited standalone predictive performance. Further studies are needed to determine whether their addition to established clinical prediction models provides incremental prognostic value.

Although the exact biological significance of these metabolic alterations remains incompletely understood, the present findings are consistent with the hypothesis that purine degradation pathways may reflect interactions between ATP depletion, oxidative stress, mitochondrial dysfunction, and endogenous antioxidant defense mechanisms described in previous experimental and clinical studies. The use of validated LC–MS/MS quantification together with prospective clinical and functional assessment represents an important strength of the study and allowed for a more detailed evaluation of these metabolic changes.

Given the exploratory nature of the study and the relatively moderate sample size, the present findings should be interpreted cautiously. Nevertheless, they suggest that purine metabolism biomarkers may represent promising complementary tools for improving our understanding of ischemic stroke pathophysiology and neurological recovery. Further multicenter studies with larger patient cohorts and longitudinal metabolic assessment are needed to better clarify their potential clinical and prognostic relevance.

In conclusion, serum uric acid, xanthine, and hypoxanthine showed distinct associations with clinical severity and functional outcome after acute ischemic stroke in unadjusted analyses. However, after multivariable adjustment, admission NIHSS remained the principal independent predictor of unfavorable 3-month functional outcome, whereas the purine biomarkers did not retain independent prognostic significance. These findings support further investigation of purine metabolism as a biologically relevant and potentially complementary biomarker pathway, but they do not establish independent clinical prognostic utility or causal mechanisms.

## Figures and Tables

**Figure 2:**
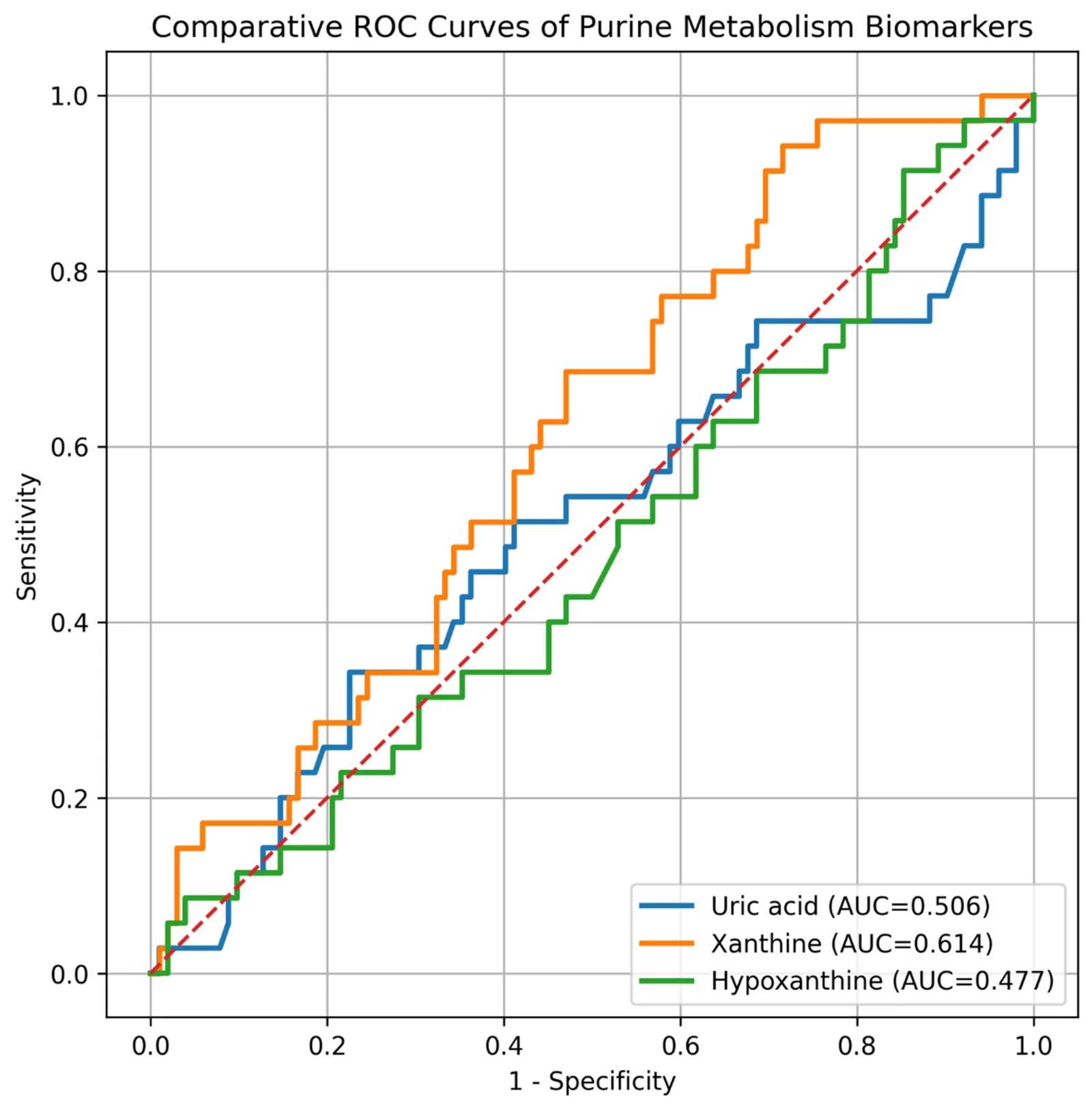
Comparative receiver operating characteristic (ROC) curves of purine metabolism biomarkers for 3-month functional outcome after acute ischemic stroke. ROC analysis was performed for serum uric acid, xanthine, and hypoxanthine concentrations. Poor functional outcome was defined as an mRS score of 4–6 at 3 months. The biomarkers were analyzed individually as continuous variables. The AUC was 0.614 for xanthine, 0.506 for uric acid, and 0.477 for hypoxanthine. The diagonal dashed line represents the reference line for no discrimination (AUC = 0.50).

**Figure 3:**
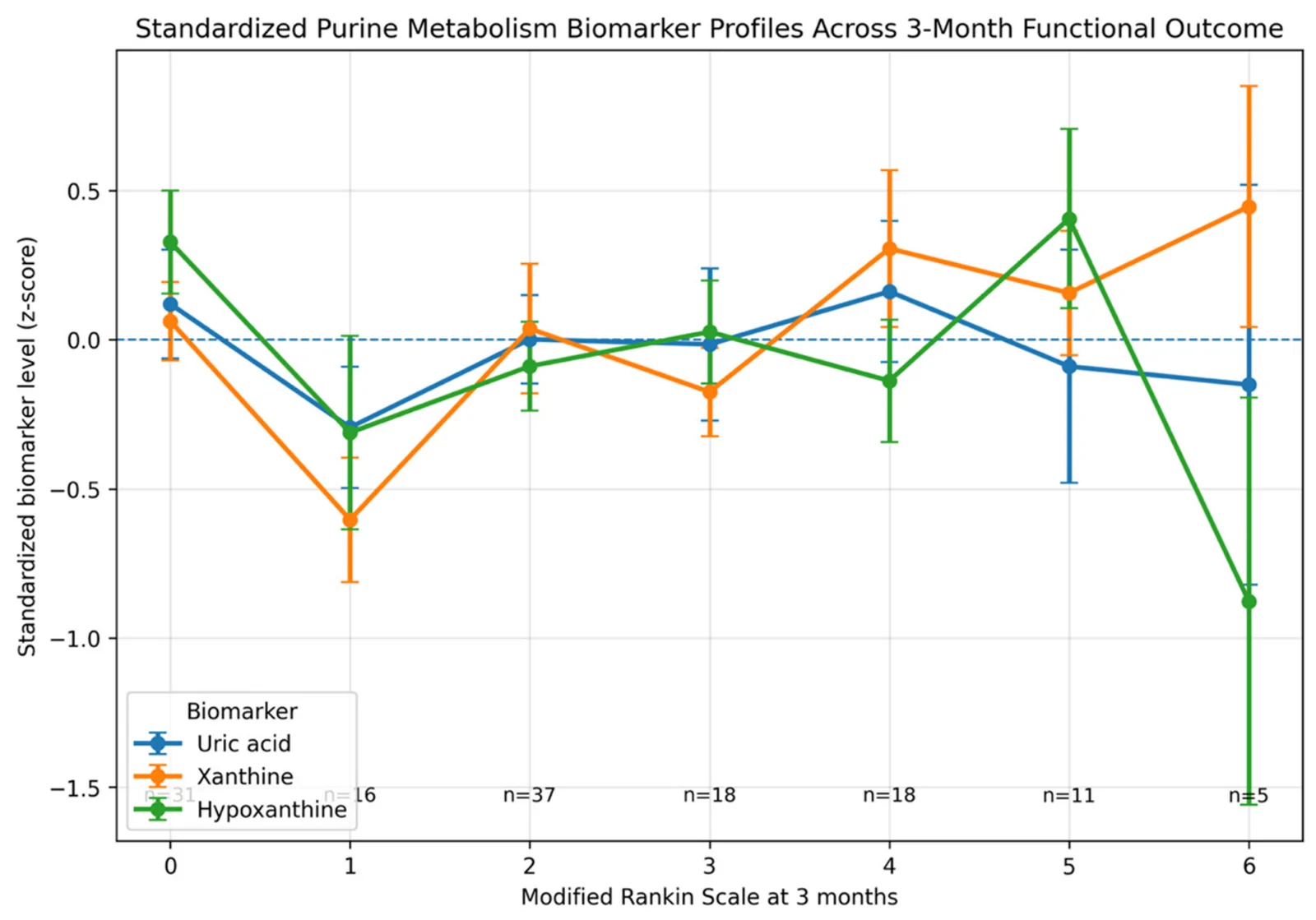
Standardized Purine Metabolism Biomarker Profiles According to Functional Outcome at 3 Months Following Acute Ischemic Stroke. Comparative analysis of standardized serum uric acid, xanthine, and hypoxanthine concentrations according to modified Rankin Scale (mRS) scores assessed at 3-month follow-up after acute ischemic stroke. Biomarker concentrations were standardized using z-score normalization in order to allow for direct intermarker comparison despite differences in concentration scales and biological ranges. Due to marked right-skewed distribution and presence of extreme outlier values, serum xanthine and hypoxanthine concentrations were logarithmically transformed prior to standardization. Mean standardized biomarker values are represented across each mRS category, while vertical error bars indicate standard error of the mean (SEM). Increasing functional disability appeared to be associated with progressively elevated xanthine concentrations, whereas serum uric acid demonstrated a tendency toward lower values in patients with poorer neurological outcome. The figure illustrates differences in measured purine metabolite concentrations according to functional outcome after acute ischemic stroke.

**Figure 4:**
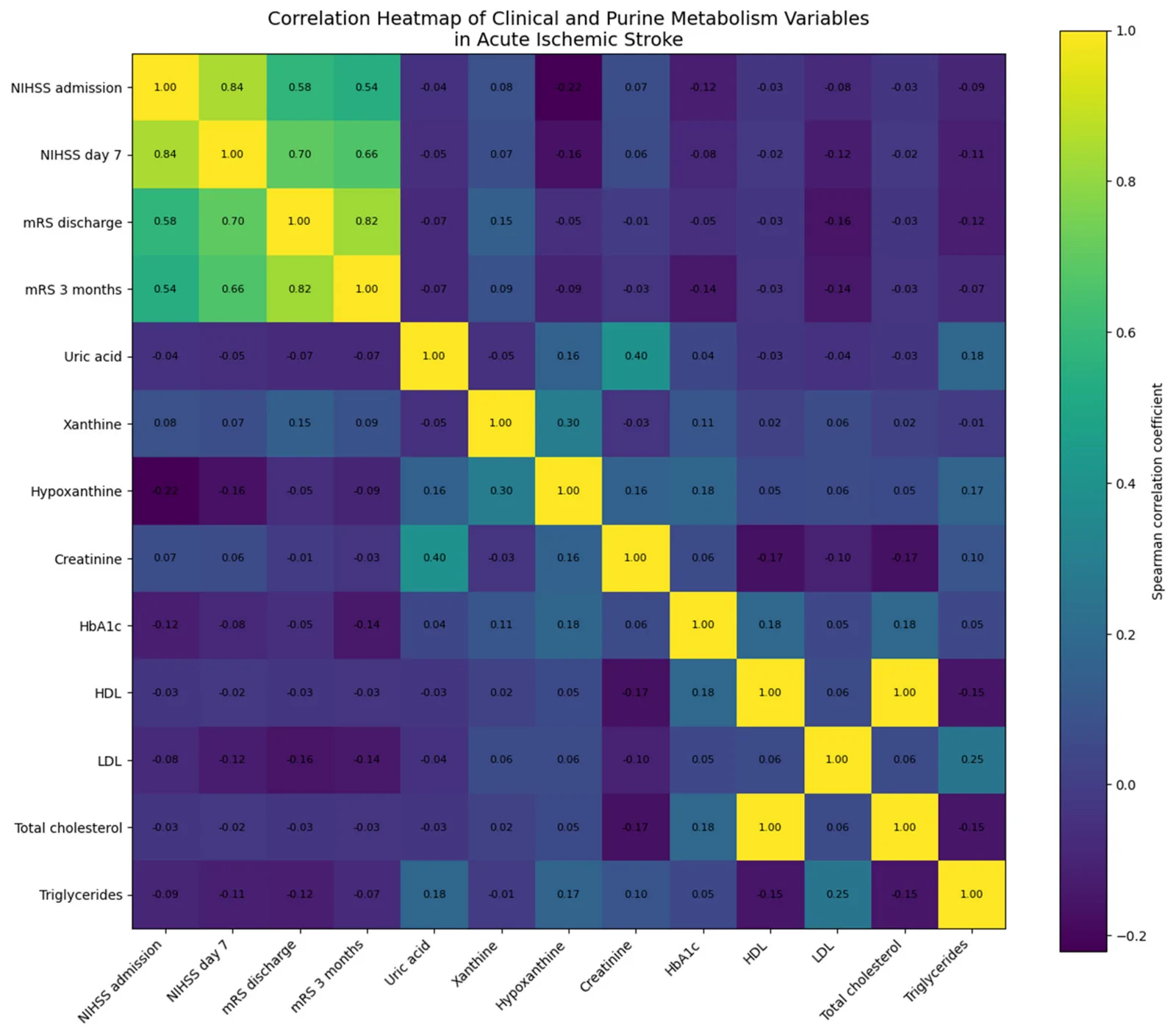
**Spearman correlation heatmap of clinical, functional, metabolic, and purine metabolism variables in acute ischemic stroke.** The heatmap displays Spearman correlation coefficients between neurological severity measures, functional outcome scores, metabolic and renal parameters, lipid variables, and purine metabolism biomarkers. Correlation coefficients are shown numerically and color-coded according to the direction and magnitude of the correlation. Spearman correlation analysis was used because several variables, including xanthine, hypoxanthine, NIHSS, and modified Rankin Scale scores, showed non-normal distributions. Positive correlations were observed among admission NIHSS, NIHSS at 7 days, mRS at discharge, and mRS at 3 months. Serum uric acid showed a positive correlation with creatinine. Xanthine and hypoxanthine showed generally weak correlations with the conventional metabolic and lipid variables.

**Figure 5:**
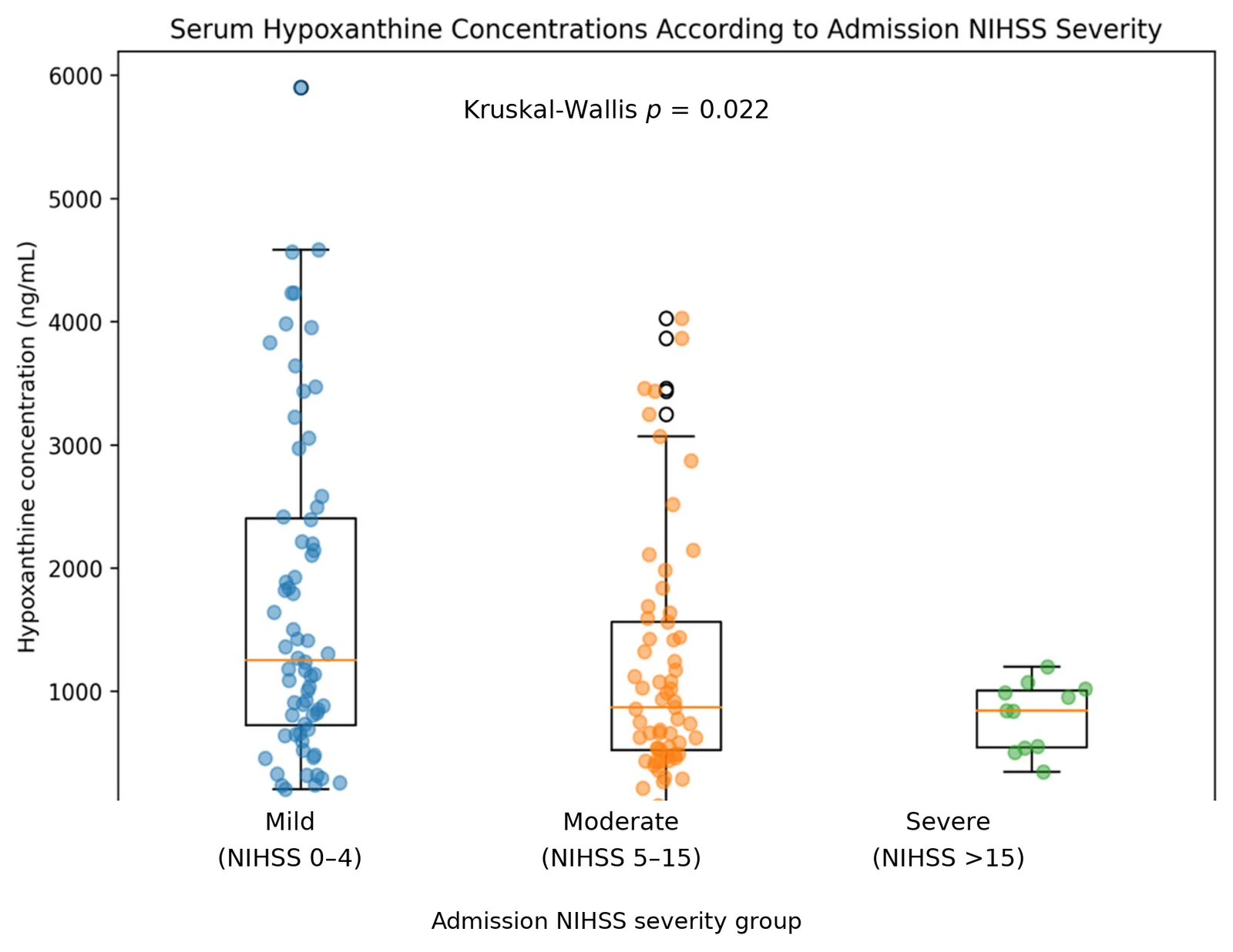
**Serum Hypoxanthine Concentrations According to Admission NIHSS Severity Categories in Acute Ischemic Stroke.** Boxplot distribution of serum hypoxanthine concentrations in patients with mild stroke (NIHSS 04), moderate stroke (NIHSS 5–15), and severe stroke (NIHSS > 15). The central line represents the median, the box represents the interquartile range, and the whiskers extend to values within 1.5 × IQR. Individual measurements are shown to illustrate data dispersion and outliers. Because hypoxanthine concentrations were right-skewed, differences between severity groups were assessed using the Kruskal–Wallis test. Hypoxanthine concentrations differed significantly between groups (*p* = 0.022), with lower values observed in patients with more severe neurological deficits. The severe-stroke subgroup contained relatively few patients, and the findings should therefore be interpreted cautiously.

**Figure 6:**
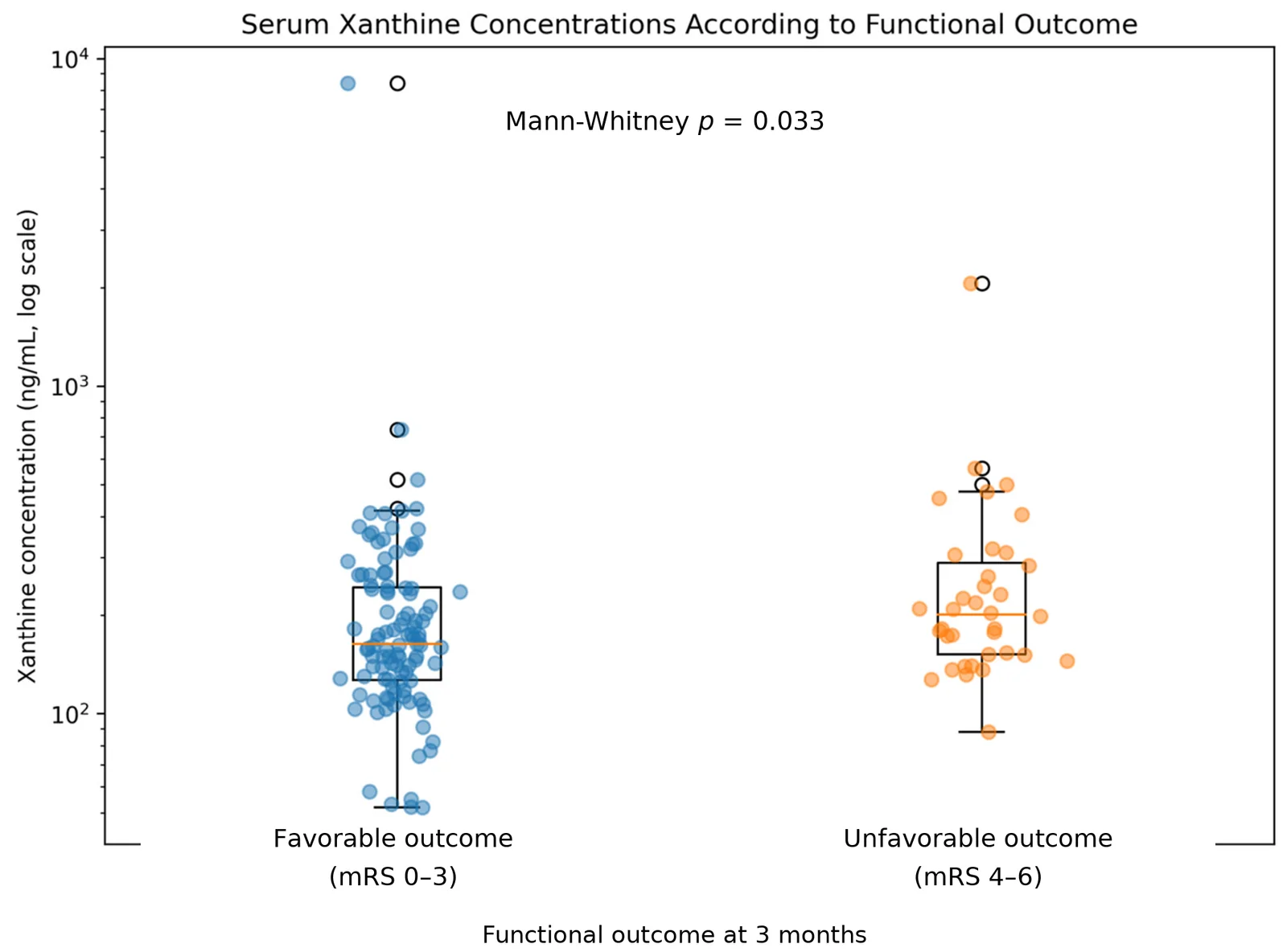
**Serum Xanthine Concentrations According to Functional Outcome at 3 Months Following Acute Ischemic Stroke.** Boxplot distribution of serum xanthine concentrations in patients with favorable outcome (mRS 0–3) and unfavorable outcome (mRS 4–6). The central line represents the median, the box represents the interquartile range, and the whiskers extend to values within 1.5 × IQR. Individual measurements are shown to illustrate data dispersion and outliers. Because xanthine concentrations were markedly right-skewed, the *y*-axis is displayed on a logarithmic scale. Differences between outcome groups were assessed using the Mann–Whitney U test. Xanthine concentrations differed significantly between groups (*p* = 0.033), with higher values observed in patients with unfavorable outcomes. Findings should be interpreted cautiously because of the exploratory analysis and the presence of extreme biomarker values.

**Figure 7:**
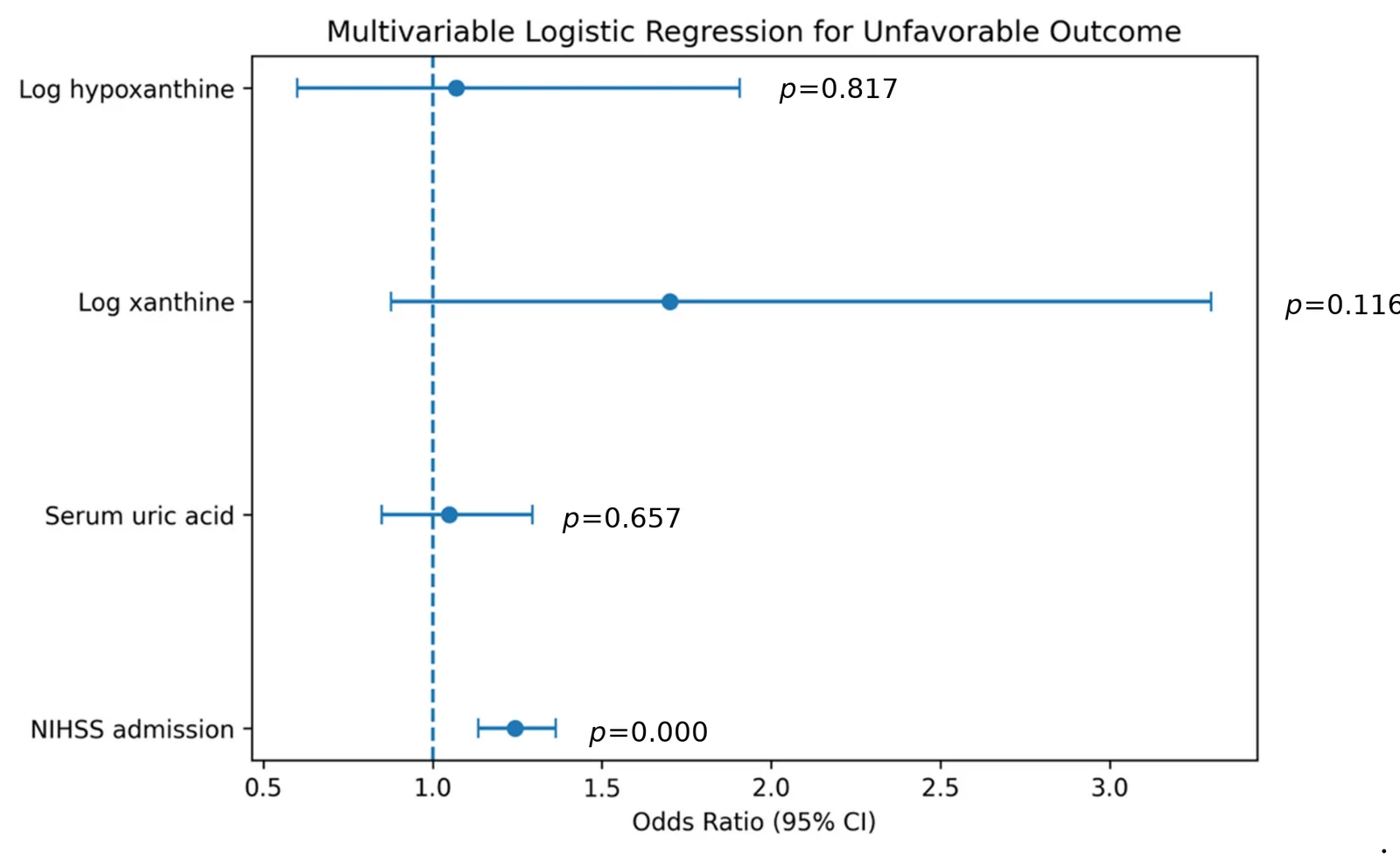
Multivariable Logistic Regression Analysis for Prediction of Unfavorable Functional Outcome Following Acute Ischemic Stroke. The Forest plot presents odds ratios (ORs) and 95% confidence intervals from the adjusted logistic regression model. Poor functional outcome was defined as an mRS score of 4–6 at 3 months. The model included admission NIHSS score, serum uric acid, log-transformed xanthine, log-transformed hypoxanthine, age, sex, hypertension, LDL cholesterol, and HDL cholesterol. Xanthine and hypoxanthine were logarithmically transformed because of their right-skewed distributions. The vertical dashed line indicates an OR of 1.0. Estimates are presented as adjusted associations and should not be interpreted as evidence of causality. No relevant multicollinearity was identified among the predictors; all variance inflation factor values were below 1.3. A complete assessment of multicollinearity, including the correlation heatmap and individual VIF values, is provided in the Supplementary Materials.

**Figure 8:**
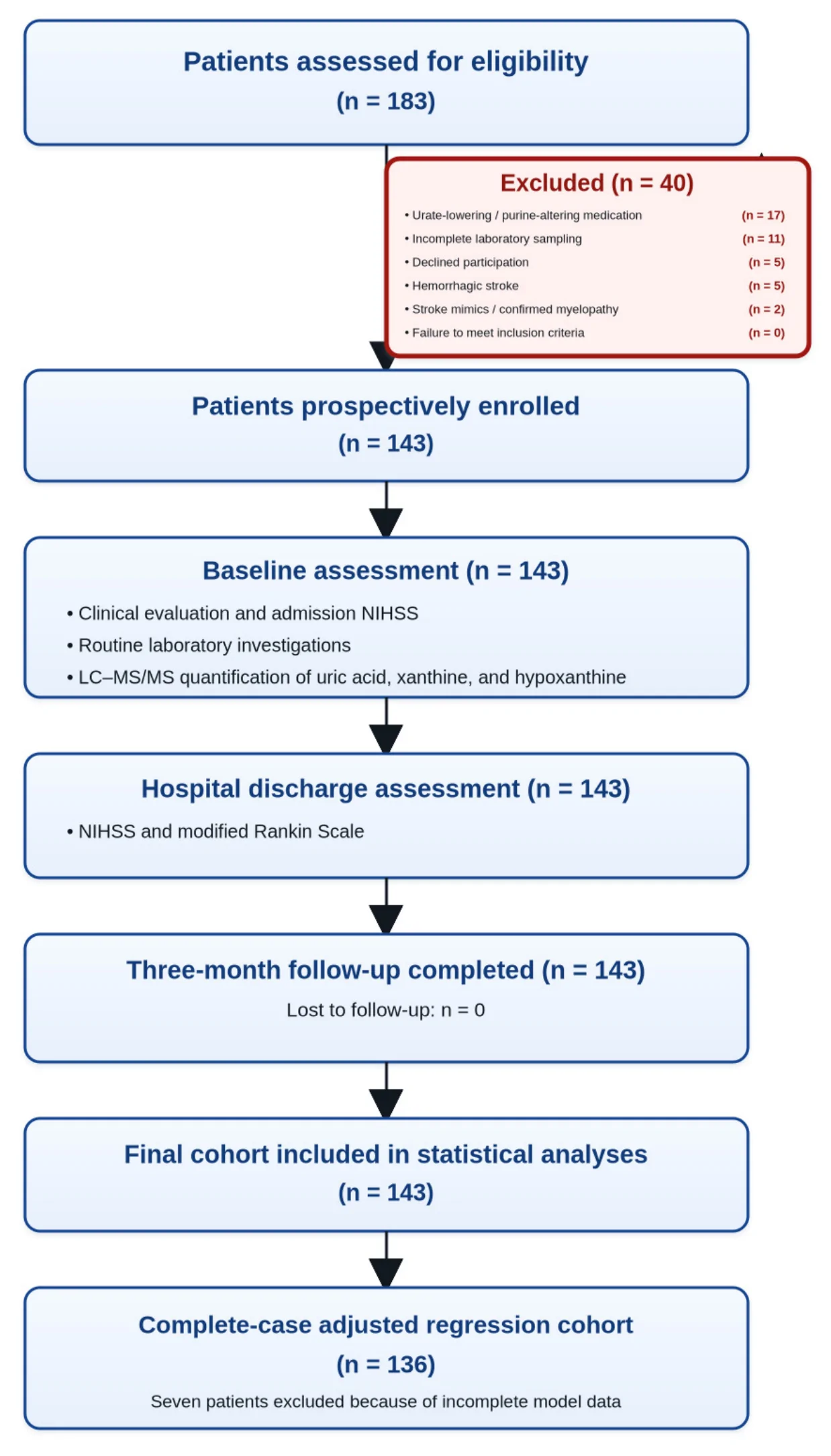
**Study flow diagram.** A total of 183 patients were assessed, 40 were excluded, and 143 were prospectively enrolled. All 143 enrolled participants completed the 3-month follow-up. The complete-case adjusted regression analysis included 136 patients, while 7 patients were excluded because of incomplete model data.

**Figure 9:**
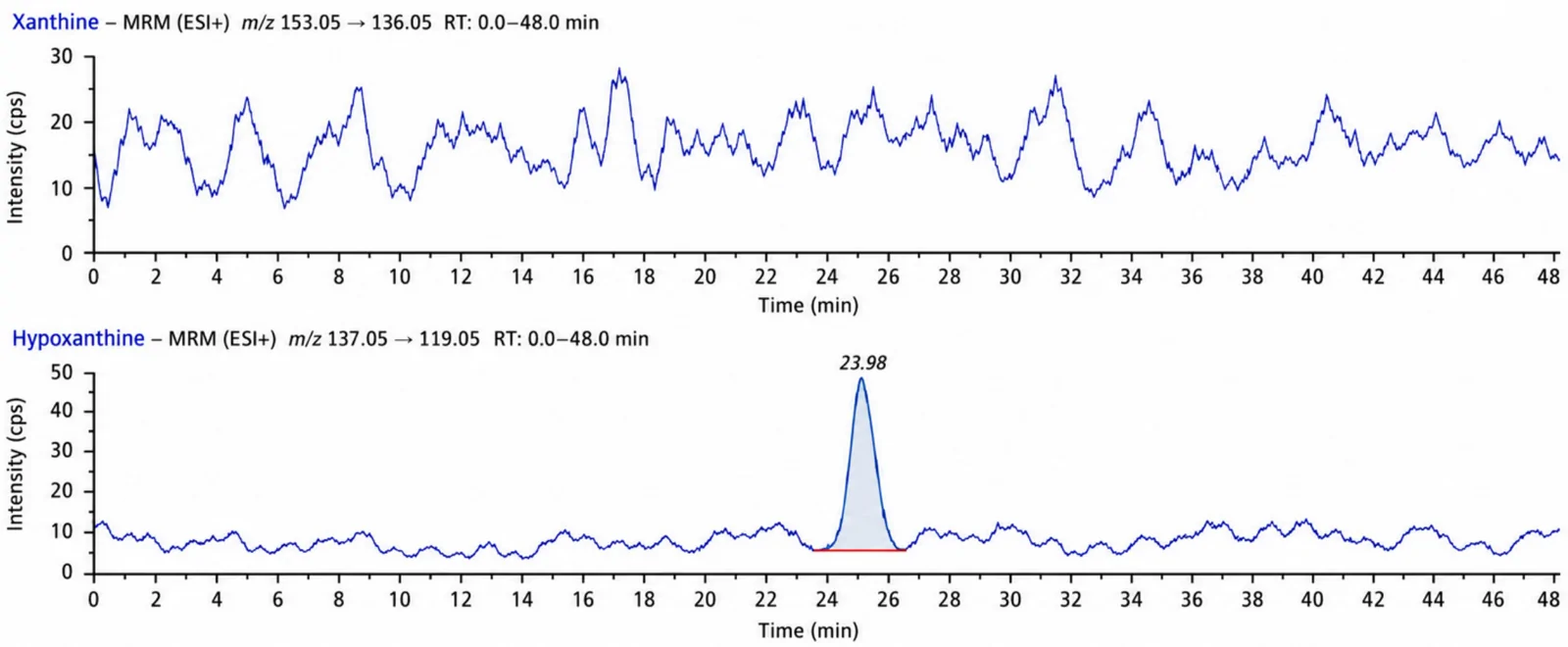
Representative LC–MS/MS Chromatograms Demonstrating Quantitative Detection of Xanthine and Hypoxanthine in Plasma Samples. Representative liquid chromatography–tandem mass spectrometry (LC–MS/MS) chromatograms illustrating analytical detection and chromatographic separation of purine metabolism intermediates in human plasma samples obtained from patients with acute ischemic stroke. The upper panel demonstrates the multiple reaction monitoring (MRM) chromatographic trace for xanthine detected in positive electrospray ionization mode (ESI+), while the lower panel illustrates the corresponding chromatographic trace for hypoxanthine, demonstrating a distinct retention peak with minimal background interference and high analytical specificity. Quantitative determination was performed using validated LC–MS/MS methodology with isotopically labeled internal standards, predefined calibration curves, and optimized chromatographic conditions. The analytical method demonstrated excellent linearity, reproducibility, and recovery across the validated concentration range and constituted the analytical basis for subsequent statistical evaluation of purine metabolism biomarkers in relation to stroke severity and functional outcome.

**Table 1:** **Baseline Descriptive Statistics of Clinical, Biochemical, and Purine Continuous variables are presented as mean ± standard deviation (SD), median, and minimum–maximum range.** Clinical severity was assessed using the National Institutes of Health Stroke Scale (NIHSS), while functional outcome was evaluated using the modified Rankin Scale (mRS). Biochemical analysis included metabolic, renal, lipid, and purine metabolism parameters, including serum uric acid, xanthine, and hypoxanthine concentrations measured during the acute phase of ischemic stroke. The number of available observations is indicated for variables with missing data; missing values resulted from incomplete clinical documentation, unavailable routine laboratory measurements, or insufficient biological samples for LC–MS/MS analysis. Sex distribution was 73 male (51.0%) and 70 female (49.0%) patients.

SEX	N	Male	Female	
	143	73 (51%)	70 (49%)	
Variable	N	Mean ± SD	Median	Min–Max
NIHSS at admission	143	6.43 ± 6.57	5.0	0–42
NIHSS at 7 days	143	5.45 ± 7.05	4.0	0–42
mRS at discharge	143	2.32 ± 1.61	2.0	0–6
mRS at 3 months	143	2.19 ± 1.72	2.0	0–6
Creatinine (mg/dL)	143	1.15 ± 0.82	0.90	0.46–5.10
HDL cholesterol (mg/dL)	141	51.69 ± 17.19	49.0	10–151
LDL cholesterol (mg/dL)	143	106.52 ± 40.90	96.0	11–222
Total cholesterol (mg/dL)	143	173.75 ± 47.70	167.0	72–325
Serum uric acid (mg/dL)	143	6.41 ± 2.11	6.38	2.19–12.00
Admission glucose (mg/dL)	143	140.40 ± 37.62	132.0	70–331
HbA1c (%)	143	7.05 ± 2.21	6.80	5.00–31.00
Urea (mg/dL)	143	69.50 ± 48.36	52.0	13–200
Age (years)	143	71.56 ± 12.33	73.0	22–95
Triglycerides (mg/dL)	137	137.51 ± 88.88	116.0	41–635
Xanthine concentration (ng/mL)	136	281.62 ± 729.25	174.44	51.88–8419.45
Hypoxanthine concentration (ng/mL)	132	1319.05 ± 1093.13	966.99	75.17–5902.93

**Note:** The total cohort comprised 143 patients; however, the number of observations varied between variables because some measurements were unavailable or could not be reliably extracted from the clinical records. The available sample sizes were reported separately for each variable. Missingness was mainly related to incomplete documentation, unavailable routine laboratory results, or insufficient biological samples for specialized LC–MS/MS measurements. Therefore, analyses involving age, HDL-cholesterol, triglycerides, xanthine, and hypoxanthine were performed using the available observations for each variable. Variable-specific sample sizes are reported because of missing or unavailable observations. Percentages and statistical analyses were calculated using the available observations for each variable.

**Table 2:** **Normality Testing**. Normality assessment of modified Rankin Scale (mRS) values at 3 months was performed separately for each xanthine quartile using the Shapiro–Wilk test. .

Quartile	Shapiro–Wilk *p*-Value
Q1	0.002
Q2	<0.001
Q3	0.030
Q4	0.002

Kruskal–Wallis Analysis of Modified Rankin Scale Scores at 3 Months Across Xanthine Quartiles. Patients were stratified into quartiles according to serum xanthine concentration. Because modified Rankin Scale (mRS) values demonstrated non-normal distribution on Shapiro–Wilk testing, non-parametric analysis was performed using the Kruskal–Wallis test. Patients were initially stratified into four quartiles (Q1–Q4) according to serum xanthine concentration measured during the acute phase of ischemic stroke. The Shapiro–Wilk test was selected due to its high sensitivity for detecting deviations from normal distribution in clinical datasets with moderate sample size. A *p*-value < 0.05 was considered indicative of non-normal distribution. Because all quartiles demonstrated statistically significant deviation from Gaussian distribution, the dataset was classified as non-parametric, supporting the subsequent use of non-parametric comparative statistical methods.

**Table 3:** **Descriptive Statistics by Xanthine Quartile.**

Xanthine Quartile	n	Mean mRS ± SD	Median mRS
Q1	36	1.75 ± 1.44	2
Q2	36	2.17 ± 1.64	2
Q3	35	2.60 ± 1.75	2
Q4	36	2.25 ± 1.98	2

Patients were categorized into quartiles according to serum xanthine concentration in order to evaluate potential associations between purine metabolism and long-term functional outcome following acute ischemic stroke. Descriptive statistical analysis was subsequently performed for modified Rankin Scale (mRS) scores assessed at three-month follow-up. Continuous variables are presented as mean ± standard deviation (SD), while median values were additionally reported due to the ordinal nature and non-normal distribution of mRS scores.

**Table 4:** Kruskal–Wallis Analysis. A comparative analysis of modified Rankin Scale (mRS) scores at three months across xanthine quartiles was conducted using the Kruskal–Wallis test.

Test	Statistic (H)	*p*-Value
Kruskal–Wallis	4.33	0.228

This non-parametric test was chosen because the mRS is an ordinal scale and normality testing showed significant deviation from a Gaussian distribution across quartile groups. The Kruskal–Wallis test assesses whether median distributions differ significantly among multiple independent groups without assuming a normal distribution of the underlying data. Statistical significance was set at *p* < 0.05. The *p*-value indicated no statistically significant differences in long-term functional outcomes among patients stratified by serum xanthine concentration quartiles.

**Table 5:** **Comparison of Serum Uric Acid Concentration According to Admission NIHSS Severity.**

NIHSS Group	n	Mean ± SD (mg/dL)	Median	Min–Max	*p*-Value
Mild stroke (NIHSS ≤ 4)	68	6.63 ± 2.15	6.56	2.19–12.00	0.289
Moderate-to-severe stroke (NIHSS > 4)	75	6.21 ± 2.07	6.31	2.34–10.60	0.289

Patients were stratified by admission NIHSS score into mild stroke (NIHSS ≤ 4) and moderate-to-severe stroke (NIHSS > 4) groups. Serum uric acid concentration was analyzed as a continuous variable. Normality testing with the Shapiro–Wilk test showed a non-normal distribution in at least one subgroup (mild stroke *p* = 0.234; moderate-to-severe stroke *p* = 0.063); therefore, a non-parametric Mann–Whitney U test was used for comparison. Continuous variables are presented as mean ± standard deviation (SD), along with the median and minimum–maximum ranges. No statistically significant difference in serum uric acid concentration was observed between NIHSS severity groups (*p* = 0.289).

**Table 6:** **Association Between Serum Uric Acid Concentration and Functional Outcome Following Acute Ischemic Stroke.** Serum uric acid concentrations were compared across mRS categories at discharge and at 3 months using the Kruskal–Wallis test.

Outcome Variable	Distribution Type	Statistical Test	Test Statistic	*p*-Value
mRS at discharge	Non-Parametric	Kruskal–Wallis	H = 5.709	0.457
mRS at 3 months	Non-Parametric	Kruskal–Wallis	H = 3.599	0.731

Serum uric acid concentrations were compared across mRS categories at discharge and at 3 months. Because mRS is an ordinal outcome, the Kruskal–Wallis test was used. No significant differences were observed at discharge (H = 5.709, *p* = 0.457) or at 3 months (H = 3.599, *p* = 0.731). Statistical significance was set at *p* < 0.05.

**Table 7:** **Xanthine and Hypoxanthine Concentrations According to Admission NIHSS Severity.** Comparative analysis of serum xanthine and hypoxanthine concentrations according to stroke severity stratified by admission NIHSS score. Patients were stratified according to admission National Institutes of Health Stroke Scale (NIHSS) scores into three predefined severity categories: mild stroke (NIHSS 0–4), moderate stroke (NIHSS 5–15), and severe stroke (NIHSS > 15). Serum xanthine and hypoxanthine concentrations were analyzed as continuous variables in order to evaluate their relationship with acute neurological severity. Distribution analysis demonstrated marked deviation from normality and substantial right-skewness for both biomarkers, particularly due to the presence of extreme outlier values. Consequently, comparative analysis between NIHSS severity groups was performed using non-parametric methodology. Continuous variables are presented as mean ± standard deviation (SD), together with median and minimum–maximum ranges in order to better characterize the highly heterogeneous biomarker distribution. Hypoxanthine concentrations were lower in patients with more severe neurological deficits at admission, whereas xanthine concentrations showed greater interindividual variability and did not differ significantly between NIHSS severity groups.

Biomarker	NIHSS Severity Group	n	Mean ± SD	Median	Min–Max	*p*-Value
Xanthine (ng/mL)	Mild (0–4)	68	317.36 ± 1002.62	164.98	54.98–8419.45	0.145
Xanthine (ng/mL)	Moderate (5–15)	64	233.88 ± 258.40	174.60	51.88–2061.39	0.145
Xanthine (ng/mL)	Severe (>15)	11	268.71 ± 131.17	195.39	136.12–500.75	0.145
Hypoxanthine (ng/mL)	Mild (0–4)	68	1717.35 ± 1330.89	1249.36	206.70–5902.93	0.022
Hypoxanthine (ng/mL)	Moderate (5–15)	61	1202.87 ± 971.80	865.48	75.17–4026.33	0.022
Hypoxanthine (ng/mL)	Severe (>15)	11	803.46 ± 275.97	843.09	344.92–1197.72	0.022

**Table 8:** Comparison of Purine Metabolism Biomarkers According to TOAST Classification. Normality testing demonstrated non-Gaussian distribution for xanthine and hypoxanthine concentrations across TOAST etiological subgroups, while serum uric acid demonstrated substantial interindividual variability. Consequently, non-parametric comparative analysis was performed using the Kruskal–Wallis test for independent samples. Continuous variables are presented as mean ± standard deviation (SD) together with median values. Although numerical differences in biomarker concentrations were observed between TOAST etiological categories, comparative analysis did not demonstrate statistically significant intergroup differences.

Biomarker	TOAST Subtype	n	Mean ± SD	Median	*p*-Value
Uric acid	Atherothrombotic	46	6.05 ± 2.38	5.59	0.256
Uric acid	Cardioembolic	30	6.71 ± 2.24	6.73	0.256
Uric acid	ESUS	31	6.25 ± 1.74	6.30	0.256
Uric acid	Small Vessel	33	6.86 ± 1.93	7.50	0.256
Xanthine	Atherothrombotic	46	209.50 ± 114.17	169.65	0.570
Xanthine	Cardioembolic	30	524.91 ± 1532.77	174.41	0.570
Xanthine	ESUS	31	245.08 ± 139.63	233.99	0.570
Xanthine	Small Vessel	33	187.96 ± 80.64	161.49	0.570
Hypoxanthine	Atherothrombotic	45	1222.93 ± 923.12	949.11	0.889
Hypoxanthine	Cardioembolic	30	1388.98 ± 1042.38	1215.41	0.889
Hypoxanthine	ESUS	30	1569.58 ± 1397.74	919.38	0.889
Hypoxanthine	Small Vessel	32	1342.82 ± 1121.63	950.38	0.889

**Table 9:** **Nutritional and Metabolic Characteristics of the Cohort.** Data are presented as mean ± standard deviation or median (interquartile range) according to variable distribution. Plasma xanthine and hypoxanthine concentrations are expressed as median (IQR) because of non-normal distribution. The table summarizes the metabolic and purine-related biomarkers measured in the study cohort and provides descriptive clinical context; it does not directly demonstrate oxidative stress, ischemic injury mechanisms, or causal associations.

Variable	Overall Cohort (n = 143)	Metabolic/Nutritional Significance
Serum Uric Acid (mg/dL)	6.41 ± 2.11	Major endogenous antioxidant and end-product of purine metabolism
Fasting Glucose (mg/dL)	140.40 ± 37.62	Acute metabolic response and glycemic status [45]
HbA1c (%)	7.05 ± 2.21	Long-term glucose regulation [46]
HDL Cholesterol (mg/dL)	51.69 ± 17.19	Protective lipid fraction [47]
LDL Cholesterol (mg/dL)	106.52 ± 40.90	Atherosclerotic risk burden [48]
Total Cholesterol (mg/dL)	173.75 ± 47.70	Global lipid metabolic profile [49]
Serum Creatinine (mg/dL)	1.15 ± 0.82	Renal determinant of uric acid clearance [50]
Triglycerides (mg/dL)	137.51 ± 88.88	Metabolic syndrome component [51]
Plasma Xanthine (ng/mL)	174.26 (134.91–254.95)	Intermediate purine degradation metabolite and oxidative stress marker [52]
Plasma Hypoxanthine (ng/mL)	1016.85 (574.67–1852.47)	Marker of ATP depletion and tissue hypoxia [53]
Hypertension, n (%)	136 (95.1%)	Cardiometabolic risk factor [54]

## Data Availability

The data presented in this study are available from the corresponding author upon reasonable request. Certain data may not be publicly available due to patient privacy and institutional ethical restrictions.

## Supplementary Materials

The following supporting information can be downloaded at: https://www.mdpi.com/article/10.3390/ijms27198486/s1.

## Abbreviations

AIS	Acute Ischemic Stroke
ATP	Adenosine Triphosphate
AUC	Area Under the Curve
BBB	Blood–Brain Barrier
CI	Confidence Interval
ESI+	Positive Electrospray Ionization
ESUS	Embolic Stroke of Undetermined Source
HDL	High-Density Lipoprotein
HPLC	High-Performance Liquid Chromatography
IQR	Interquartile Range
LC–MS/MS	Liquid Chromatography–Tandem Mass Spectrometry
LDL	Low-Density Lipoprotein
LLOQ	Lower Limit of Quantification
mRS	Modified Rankin Scale
MRM	Multiple Reaction Monitoring
NIHSS	National Institutes of Health Stroke Scale
OR	Odds Ratio
QTOF	Quadrupole Time-of-Flight
ROC	Receiver Operating Characteristic
ROS	Reactive Oxygen Species
rtPA	Recombinant Tissue Plasminogen Activator
SD	Standard Deviation
SEM	Standard Error of the Mean
TOAST	Trial of Org 10172 in Acute Stroke Treatment
UHPLC	Ultra-High Performance Liquid Chromatography
XO	Xanthine Oxidase
