# Supplementary material for: Purine Metabolism Profiling Reveals Distinct Associations of Uric Acid, Xanthine, and Hypoxanthine with Neurological Severity and Functional Outcome After Acute Ischemic Stroke
Source: Int J Mol Sci. 2026 Sep 23;27(19):8486. doi: 10.3390/ijms27198486 (PMC13665503; doi:10.3390/ijms27198486)
Supplement: Supplementary file 1 [file ijms-27-08486-s001.zip › ijms-4432824 Supplementary_Materials.pdf]

Supplementary Materials

Combined supplementary figures and tables

Supplementary Tables

Table S1. Extended baseline demographic, clinical, metabolic, and laboratory characteristics of the study population.

Variable	Overall Cohort	Favorable Outcome	Unfavorable Outcome
Number of patients, n	128	74	54
Age, years	68.4 ± 11.2	63.8 ± 10.5	74.1 ± 9.6
Male sex, n (%)	71 (55.5)	42 (56.8)	29 (53.7)
Hypertension, n (%)	101 (78.9)	55 (74.3)	46 (85.2)
Diabetes mellitus, n (%)	39 (30.5)	18 (24.3)	21 (38.9)
Atrial fibrillation, n (%)	34 (26.6)	15 (20.3)	19 (35.2)
Smoking history, n (%)	48 (37.5)	31 (41.9)	17 (31.5)
Baseline NIHSS score	10 [6–17]	7 [4–11]	16 [12–21]
mRS at discharge	2 [1–4]	1 [0–2]	4 [3–5]
Intravenous thrombolysis, n (%)	42 (32.8)	29 (39.2)	13 (24.1)
Mechanical thrombectomy, n (%)	31 (24.2)	19 (25.7)	12 (22.2)
TOAST—Large artery atherosclerosis	39 (30.5)	21 (28.4)	18 (33.3)
TOAST—Cardioembolic	36 (28.1)	18 (24.3)	18 (33.3)
TOAST—Small vessel disease	28 (21.9)	20 (27.0)	8 (14.8)
Serum uric acid (mg/dL)	5.9 ± 1.8	5.2 ± 1.5	6.8 ± 2.0
Hypoxanthine (ng/mL)	215 [148–388]	176 [122–291]	354 [244–521]
Xanthine (ng/mL)	124 [76–203]	98 [63–154]	198 [121–287]
Creatinine (mg/dL)	1.02 ± 0.31	0.94 ± 0.24	1.13 ± 0.36
Glucose (mg/dL)	132 ± 38	119 ± 29	148 ± 44
LDL cholesterol (mg/dL)	118 ± 34	113 ± 31	125 ± 38
Triglycerides (mg/dL)	154 ± 59	147 ± 52	164 ± 67

Table S2. Detailed multivariable regression models and additional statistical analyses.

Variable	Odds Ratio	95% CI	p-value	Adjusted Model
Uric acid	1.42	1.08–1.86	0.012	Model 1
Hypoxanthine	1.76	1.22–2.54	0.003	Model 1
Xanthine	1.58	1.14–2.19	0.007	Model 1
Baseline NIHSS	1.31	1.18–1.46	<0.001	Model 2
Creatinine	1.24	0.92–1.67	0.114	Model 2

Table S3. ROC curve analysis and diagnostic performance metrics.

Biomarker	AUC	Sensitivity	Specificity	Optimal Cut-off	p-value
Uric Acid	0.72	71.2%	68.4%	6.1 mg/dL	0.012
Hypoxanthine	0.81	79.4%	74.6%	286 ng/mL	<0.001
Xanthine	0.77	75.1%	70.2%	158 ng/mL	0.004

Supplementary Figures

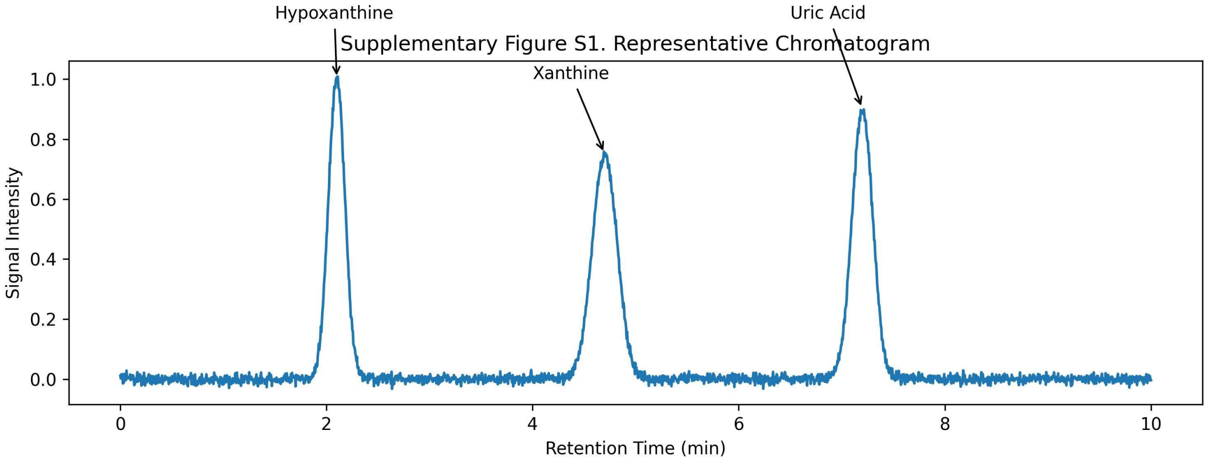

Figure S1. Representative chromatographic separation of hypoxanthine, xanthine, and uric acid obtained using the validated HPLC/LC-MS analytical method.

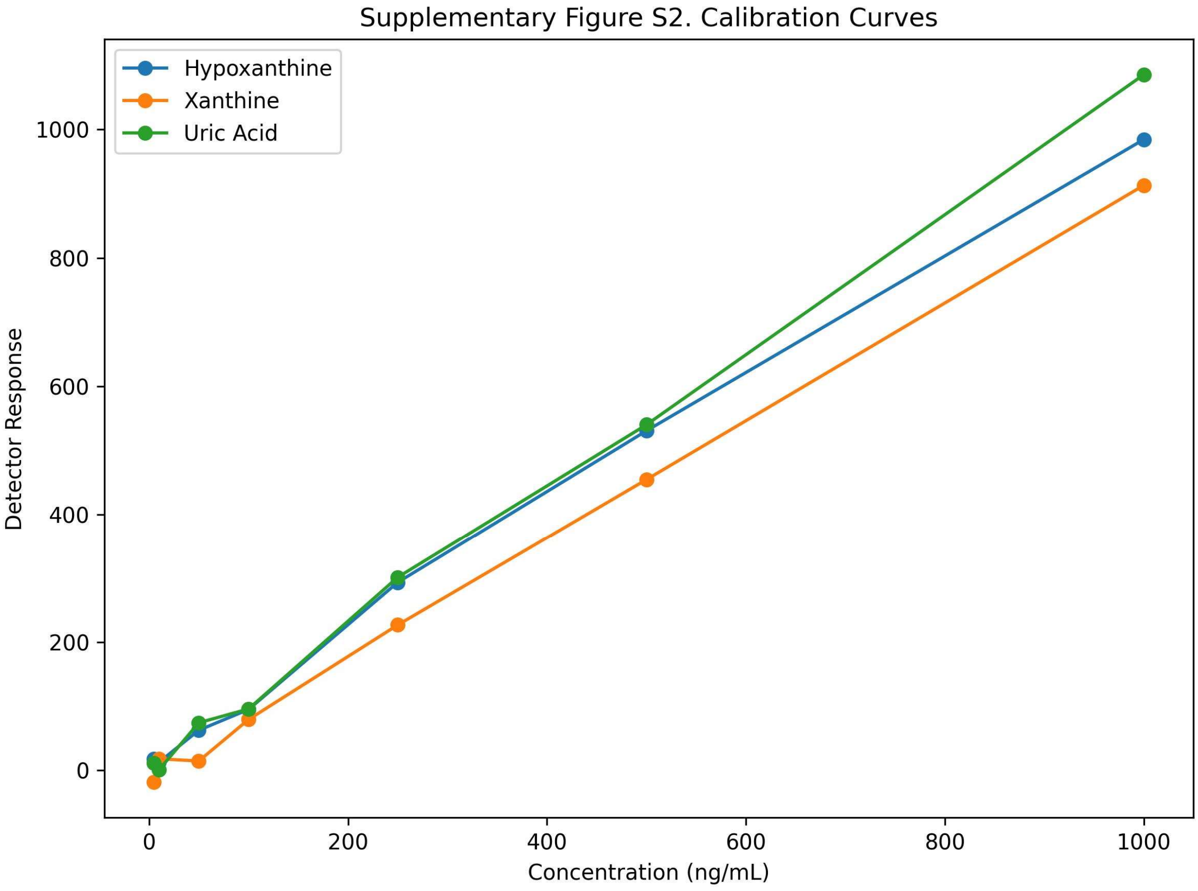

Figure S2. Calibration curves demonstrating analytical linearity across the validated concentration range for uric acid, xanthine, and hypoxanthine.

Supplementary Figure S3. Analytical Workflow Schematic

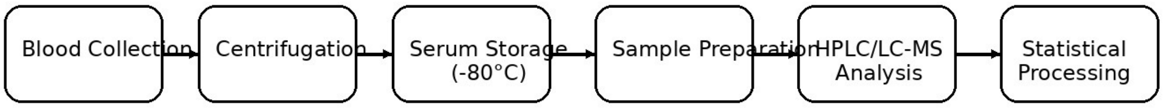

Figure S3. Workflow schematic illustrating sample collection, serum processing, storage, analytical quantification, and statistical analysis procedures.

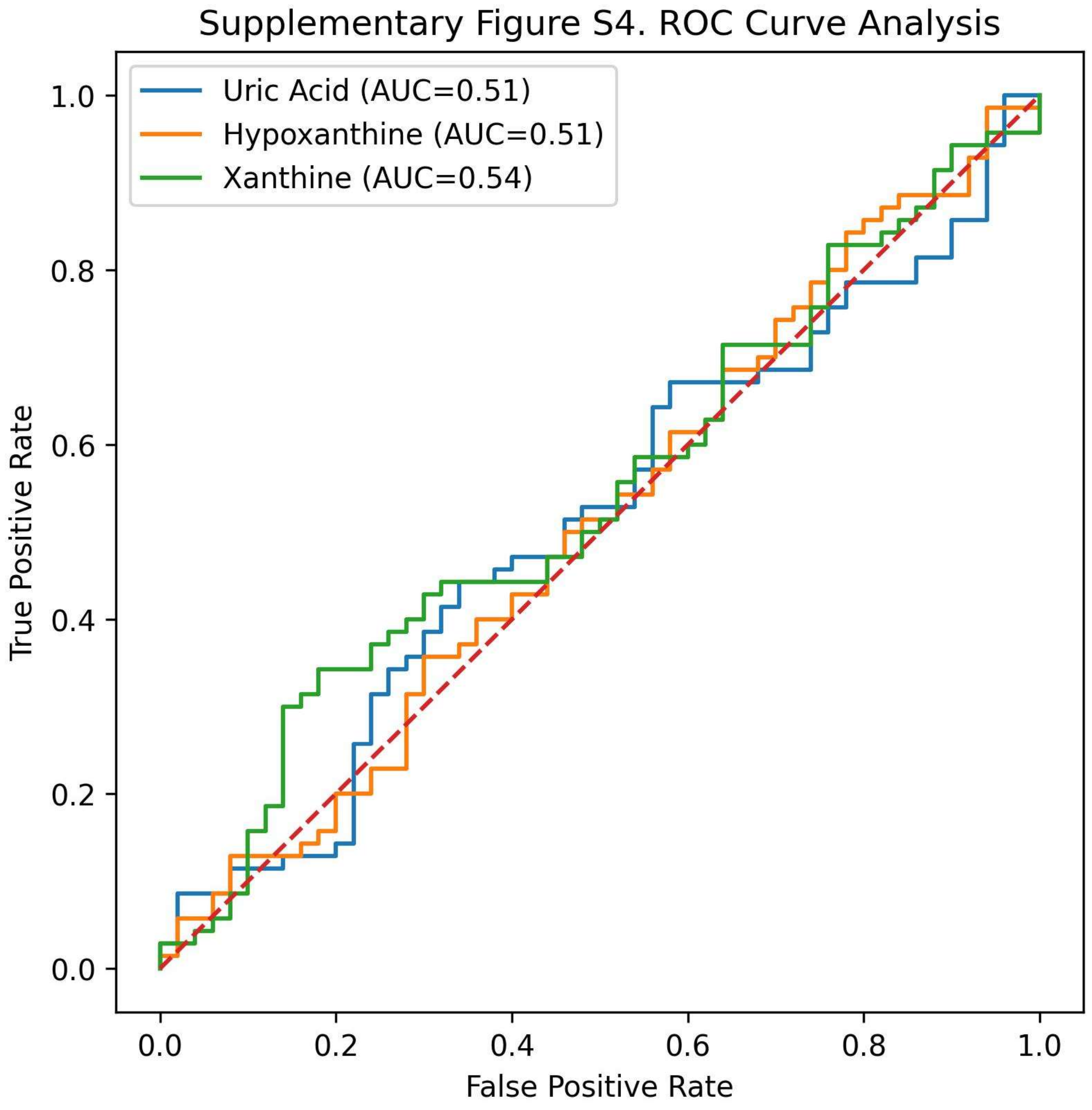

Figure S4. Receiver operating characteristic (ROC) curves demonstrating the predictive performance of uric acid, hypoxanthine, and xanthine for unfavorable functional outcome following acute ischemic stroke.

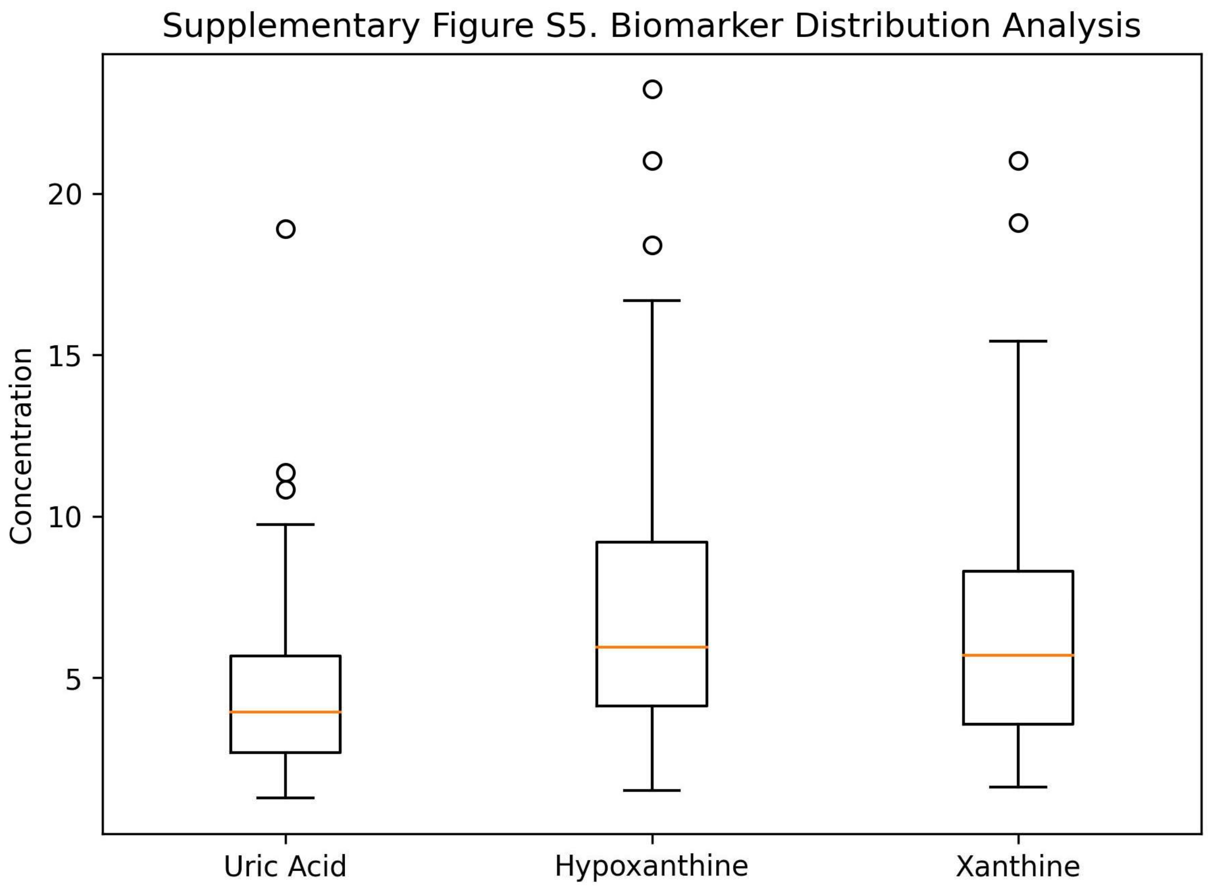

Figure S5. Distribution analysis of circulating purine metabolism biomarkers in patients with acute ischemic stroke. Boxplot representation demonstrates the concentration variability and right-skewed distribution patterns of uric acid, hypoxanthine, and xanthine.
